# Functional Specialization of the Small Interfering RNA Pathway in Response to Virus Infection

**DOI:** 10.1371/journal.ppat.1003579

**Published:** 2013-08-29

**Authors:** Joao Trindade Marques, Ji-Ping Wang, Xiaohong Wang, Karla Pollyanna Vieira de Oliveira, Catherine Gao, Eric Roberto Guimaraes Rocha Aguiar, Nadereh Jafari, Richard W. Carthew

**Affiliations:** 1 Department of Molecular Biosciences, Northwestern University, Evanston, Illinois, United States of America; 2 Department of Biochemistry and Immunology, Instituto de Ciências Biológicas, Universidade Federal de Minas Gerais, Belo Horizonte, Minas Gerais, Brazil; 3 Department of Statistics, Northwestern University, Evanston, Illinois, United States of America; 4 Genomics Core, Center for Genetic Medicine, Feinberg School of Medicine, Northwestern University, Chicago, Illinois, United States of America; University of California Riverside, United States of America

## Abstract

In *Drosophila*, post-transcriptional gene silencing occurs when exogenous or endogenous double stranded RNA (dsRNA) is processed into small interfering RNAs (siRNAs) by Dicer-2 (Dcr-2) in association with a dsRNA-binding protein (dsRBP) cofactor called Loquacious (Loqs-PD). siRNAs are then loaded onto Argonaute-2 (Ago2) by the action of Dcr-2 with another dsRBP cofactor called R2D2. Loaded Ago2 executes the destruction of target RNAs that have sequence complementarity to siRNAs. Although Dcr-2, R2D2, and Ago2 are essential for innate antiviral defense, the mechanism of virus-derived siRNA (vsiRNA) biogenesis and viral target inhibition remains unclear. Here, we characterize the response mechanism mediated by siRNAs against two different RNA viruses that infect Drosophila. In both cases, we show that vsiRNAs are generated by Dcr-2 processing of dsRNA formed during viral genome replication and, to a lesser extent, viral transcription. These vsiRNAs seem to preferentially target viral polyadenylated RNA to inhibit viral replication. Loqs-PD is completely dispensable for silencing of the viruses, in contrast to its role in silencing endogenous targets. Biogenesis of vsiRNAs is independent of both Loqs-PD and R2D2. R2D2, however, is required for sorting and loading of vsiRNAs onto Ago2 and inhibition of viral RNA expression. Direct injection of viral RNA into Drosophila results in replication that is also independent of Loqs-PD. This suggests that triggering of the antiviral pathway is not related to viral mode of entry but recognition of intrinsic features of virus RNA. Our results indicate the existence of a vsiRNA pathway that is separate from the endogenous siRNA pathway and is specifically triggered by virus RNA. We speculate that this unique framework might be necessary for a prompt and efficient antiviral response.

## Author Summary

The RNA interference (RNAi) pathway utilizes small non-coding RNAs to silence gene expression. In insects, RNAi regulates endogenous genes and functions as an RNA-based immune system against viral infection. Here we have uncovered details of how RNAi is triggered by RNA viruses. Double-stranded RNA (dsRNA) generated as a replication intermediate or from transcription of the RNA virus can be used as substrate for the biogenesis of virus-derived small interfering RNAs (vsiRNAs). Unlike other dsRNAs, virus RNA processing involves Dicer but not its canonical partner protein Loqs-PD. Thus, vsiRNA biogenesis is mechanistically different from biogenesis of endogenous siRNAs or siRNAs derived from other exogenous RNA sources. Our results suggest a specialization of the pathway dedicated to silencing of RNA viruses versus other types of RNAi silencing. The understanding of RNAi mechanisms during viral infection could have implications for the control of insect-borne viruses and the use of siRNAs to treat viral infections in humans.

## Introduction

RNA interference (RNAi) utilizes small non-coding RNAs in association with an Argonaute (Ago) protein to regulate gene expression in virtually all eukaryotes [1], [2], [3]. In animals, there are three major classes of small non-coding RNAs: microRNAs (miRNAs), piwi-interacting RNAs (piRNAs), and small interfering RNAs (siRNAs) [4]. Each small RNA class requires different enzymes for its biogenesis, and each class tends to associate with distinct Ago proteins [3]. siRNAs are made from long double stranded RNA (dsRNA) precursors derived from transposable elements, extended RNA hairpins, and sense-antisense RNA pairs [5]. Exogenous dsRNA introduced by injection or transfection can also generate siRNAs. In *Drosophila*, exogenous and endogenous dsRNAs are processed into siRNAs by Dicer-2 (Dcr-2) in association with the PD isoform of Loquacious (Loqs-PD) [6], [7]. There are four Loqs isoforms that participate in the biogenesis of distinct classes of small RNAs but only isoform PD is required for siRNA processing [8], [9], [10]. Endo-siRNAs from endogenous precursors and exo-siRNAs from exogenous precursors are then sorted by a protein complex composed of Dcr-2 and R2D2 to be loaded onto Argonaute-2 (Ago2) [6], [11]. Ago2 then ejects one strand of the siRNA duplex to generate a mature RNA-induced silencing complex (RISC) containing only the guide strand of the siRNA [12], [13]. The mature Ago2-RISC is then capable of cleaving single-stranded RNAs complementary to the guide siRNA [5].

The siRNA pathway is a major arm of the antiviral response in plants and invertebrate animals [14], [15]. In *Drosophila*, *Ago2*, *R2D2* and *Dcr-2* mutant individuals exhibit increased sensitivity to infection by several viruses [16], [17], [18], [19]. Virus-derived siRNAs (vsiRNAs) are generated in adult individuals and cell lines infected with different viruses [19], [20], [21], [22], [23], [24]. For example, *Drosophila* S2 cells infected with Flock house virus (FHV) generate 21-nucleotide (nt) vsiRNAs that preferentially map to the 5′ region of both RNA segments of the viral genome [20], [21]. Similarly, FHV-infected adults generate vsiRNAs from the positive strand of the viral genome unless a replication deficient FHV is used, in which case the vsiRNAs map to both strands [17]. This has been interpreted to suggest that Dcr-2 targets nascent dsRNA formed as intermediates of FHV genome replication [21]. Adult flies infected with Vesicular Stomatitis virus (VSV) also generate 21-nt vsiRNAs but these show no obvious bias for RNA strand or region of the genome [22]. These studies suggest that different mechanisms exist for activation of the siRNA pathway during infection with different RNA viruses.

Here, we utilize wildtype and mutant *Drosophila* to characterize the siRNA responses triggered by two RNA viruses, Sindbis virus (SINV) and VSV. SINV belongs to the *Togaviridae* family and has a positive RNA genome, while VSV belongs to the *Rhabdoviridae* family and has a negative RNA genome. We chose SINV and VSV because they have distinct strategies of replication, allowing us to uncover common and unique features of each antiviral response. Our results indicate that biogenesis of siRNAs from viral RNA is mechanistically distinct from siRNA biogenesis from endogenous or exogenous sources of dsRNA. We propose a mechanism whereby dsRNAs generated during viral replication and transcription are sources of vsiRNAs, and viral transcripts are major targets of RISC-mediated silencing.

## Results

### Antiviral defense is independent of Loqs-PD

**Figure 1:**
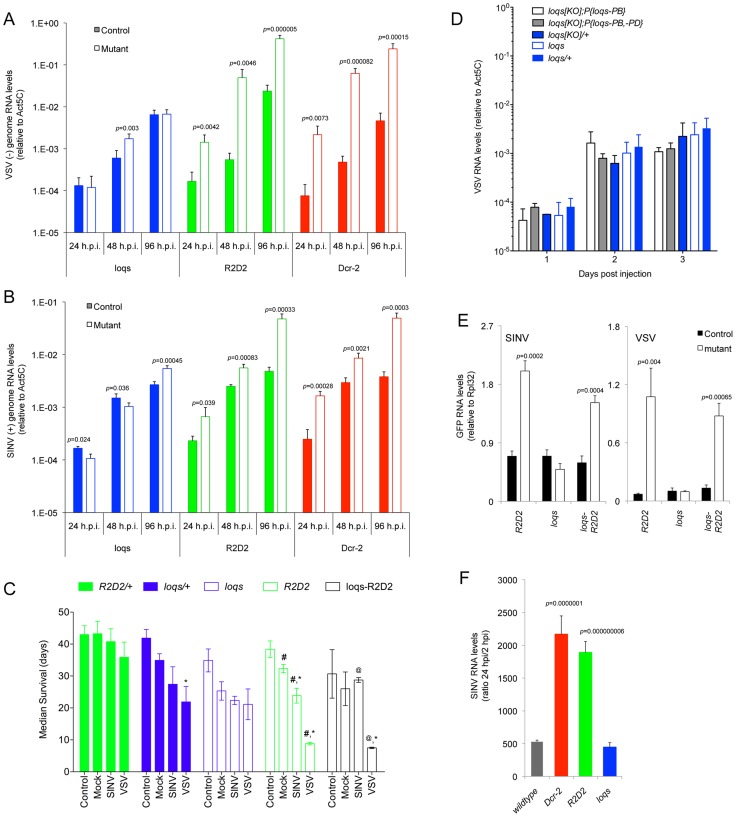
R2D2 but not Loqs-PD is required for defense against RNA viruses in *Drosophila*. (**A,B**) Viral genome RNA levels in *Dcr-2*, *R2D2* and *loqs* mutant animals (open bars) compared to their wildtype controls, *Dcr-2/+*, *R2D2/+* and *loqs/+* (closed bars). Animals were infected with VSV (**A**) or SINV (**B**) for the indicated times. *p* values below 0.05 are shown for differences between mutants and wildtype. (**C**) Median survival of control, mock-, VSV- and SINV-infected animals of the indicated genotypes. (*) indicates *p*<0.05 comparing mock- to virus-infected animals of the same genotype, (#) indicates *p*<0.05 comparing an infected mutant to its matched wildtype control, and (@) indicates *p*<0.05 comparing the *loqs R2D2* mutant to its matched *loqs* mutant. Statistical analysis of median survival is shown in Table S1 and the survival curves are shown in Fig. S1. (**D**) VSV RNA levels at various days post infection in *loqs^KO^* null mutant animals with rescue transgenes for Loqs-PB only or Loqs-PB+Loqs-PD. Also shown are *loqs* heterozygous mutants as wildtype controls and the *loqs* mutant genotype used in (**A**). (**E**) GFP RNA levels in wildtype control and mutant animals infected with recombinant VSV or SINV expressing GFP. (**F**) Fold increase of SINV RNA levels in wildtype and mutant embryos at 24 h post injection compared to 2 h post injection of purified RNA. *p* values are shown for significant differences in SINV RNA levels between wildtype and mutant.

Although Loqs-PD and R2D2 execute different steps in the endo-/exo-siRNA pathway, their roles in the antiviral siRNA pathway are less clear. To explore this issue, we infected *Drosophila* adults by injecting either SINV or VSV into their hemocoelic cavities. We monitored viral RNA genome levels for three days post-infection (dpi), and observed significantly higher levels of SINV and VSV genomes in *Dcr-2* and *R2D2* mutants, compared to wildtype (Fig. 1A,B). In contrast, *loqs* mutants showed viral genome levels indistinguishable from wildtype. We also analyzed host survival after viral infection. When wildtype adults were injected with VSV or SINV, they showed a weak reduction in survival compared to mock-injected animals (Figs. 1C and S1, Table S1). Likewise, *loqs* mutants showed a comparably weak reduction in lifespan due to VSV or SINV injection when compared to mock-injected. In contrast, *R2D2* mutants had a significantly reduced lifespan upon injection of either VSV or SINV (Figs. 1C and S1, Table S1).

The *loqs* mutants carried a null mutant allele over an allele that still has low but detectable *loqs-pd* mRNA expression [10]. It was possible that the residual Loqs-PD was sufficient to rescue the antiviral response that we had detected in the mutants. Therefore, we infected *loqs* null mutants that also carried a *loqs* transgene only expressing the Loqs-PB isoform. This transgene is able to rescue the miRNA pathway but leaves the siRNA pathway completely disabled [8]. The infected mutants displayed similar VSV RNA levels compared to wildtype (Fig. 1D). We also infected null mutants that carried a transgene expressing both Loqs-PB and Loqs-PD, which rescues both miRNA and siRNA pathways [8]. These mutants behaved similarly to the PB-only mutants (Fig. 1D). Together these results indicate that Loqs-PD is completely dispensable for inhibiting virus replication and promoting host survival after infection.

The surprisingly superfluous character of Loqs-PD suggested that there might be redundancy between R2D2 and Loqs-PD, as can happen under some circumstances [6]. Therefore, we analyzed viral infection of *loqs R2D2* double mutants. We injected recombinant viruses expressing green fluorescent protein (GFP) to facilitate the direct comparison between viruses, since GFP expression faithfully reflects replication levels for both VSV and SINV [25], [26]. In VSV or SINV infected animals, GFP expression was similarly elevated in *loqs R2D2* double mutants compared to *R2D2* single mutants (Fig. 1E). There was slightly less GFP expression in the double mutant compared to *R2D2* alone, which could suggest that Loqs-PD enhances viral replication in the absence of R2D2. Nevertheless there was no evidence of an additive effect between *loqs* and *R2D2*. We also looked at host lifespan after VSV infection, and observed that *R2D2* and *loqs R2D2* mutants showed similar lifespan reduction (Fig. 1C and S1). Although SINV infection similarly affected *R2D2* and *loqs R2D2* lifespans, this result was complicated by the reduction in lifespan already observed in mock-injected animals (Fig. 1C and S1). Since *R2D2* and *loqs R2D2* mutant animals showed similar effects on virus replication and host survival, it suggests that even in the absence of R2D2, Loqs-PD does not have an impact on viral infection.

Exogenous dsRNA, when injected into *Drosophila* cells, requires Loqs-PD to generate an RNAi response [6]. It was intriguing that viral RNA, though extrinsic to cells, does not require Loqs-PD to generate an antiviral response. We hypothesized that either intrinsic features of viral RNA, its virion packaging, its route of entry, or the nature of the infected cells could determine this Loqs-PD independence. We had previously found that injection of exogenous dsRNA into *Drosophila* embryos triggered silencing in a manner highly dependent upon Loqs-PD [6]. Therefore, we injected RNA purified from SINV virions into *Drosophila* embryos. RNA levels were measured at 24 hours post injection (hpi) and normalized to the levels detected at 2 hpi. Wildtype embryos experienced a 500-fold increase in SINV genome levels between 2 and 24 hpi (Fig. 1F), indicating that the injected RNA was competent for replication. RNA replication was strongly enhanced in *Dcr-2* and *R2D2* mutant embryos compared to wildtype. In contrast, *loqs* mutant embryos experienced replication levels that were no greater than wildtype (Fig. 1F). This result indicates that the Loqs-independent antiviral response recognizes intrinsic features of the viral RNA or its replicative forms.

### Biogenesis of vsiRNAs does not require Loqs-PD

We sequenced small RNAs from infected animals to study vsiRNA production. A time of 48 h post-infection was chosen for analysis because it is the time when viral RNA levels approach a plateau. *Dcr-2*, *R2D2* and *loqs* mutants were analyzed and compared to wildtype to characterize the roles of these genes (Tables S2 and S3). Small RNAs derived from the *Drosophila* genome were initially analyzed. As detected by RNA read density along the major autosomes, overall distributions of RNAs along the genomes of *R2D2* and *loqs* mutants were similar to wildtype (Fig. S2). As previously reported [6], [27], [28], *loqs*, *Dcr-2* and *R2D2* mutants showed a decreased abundance of specific endogenous small RNAs such as 21-nt endo-siRNAs derived from *Drosophila* mRNAs (Fig. S3).

**Figure 2:**
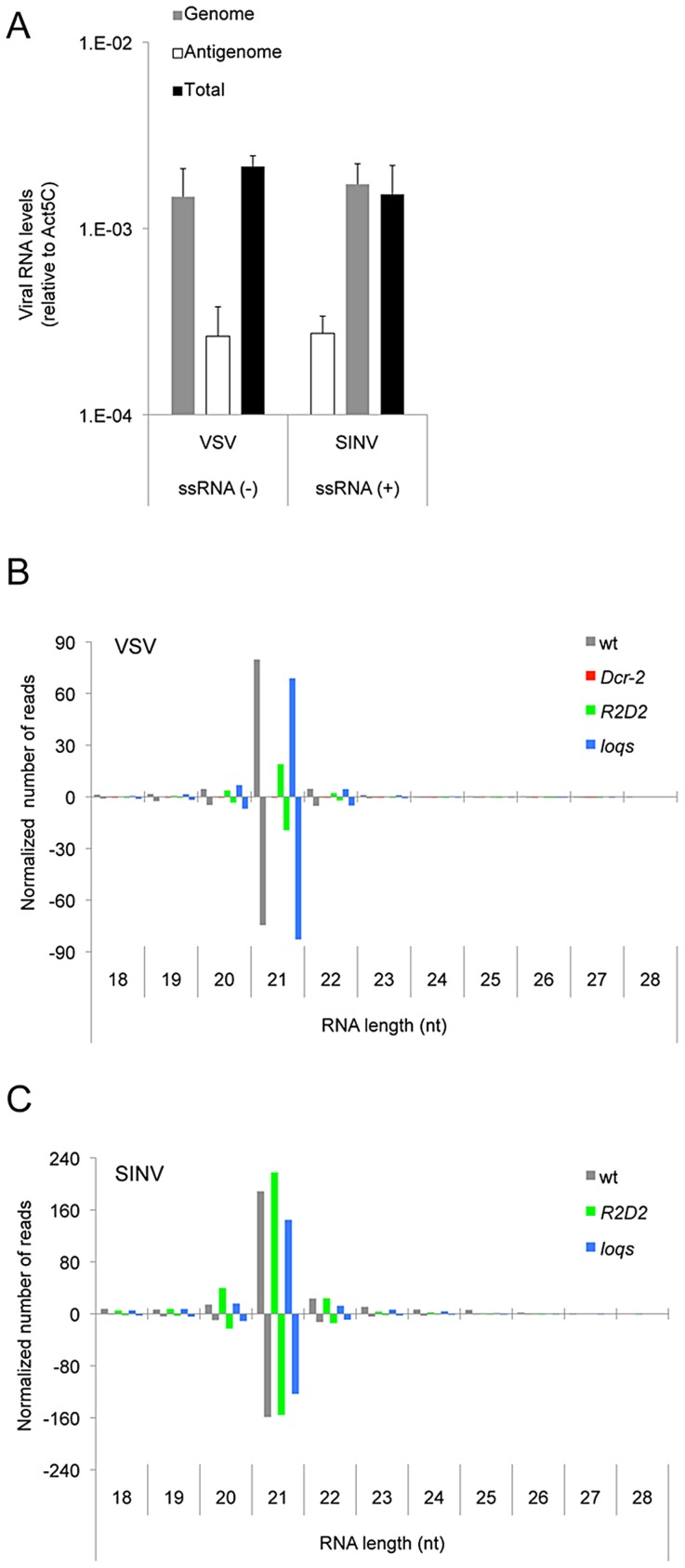
vsiRNA abundance is dependent on Dcr-2 but not Loqs-PD. (**A**) Levels of the genome RNA strand, antigenome RNA strand, and total virus RNA from VSV and SINV infected animals 48 hours post infection. The polarity of SINV and VSV genomes are indicated. (**B,C**) Normalized levels of sequenced small RNAs of different size that match the VSV (**B**) and SINV (**C**) genomes. Shown are levels after infection of wildtype (wt), *Dcr-2*, *R2D2* and *loqs* mutants. Bars above the midline denote positive-stranded small RNAs, and bars below the midline denote negative-stranded small RNAs.

SINV and VSV have single-stranded RNA genomes, and they synthesize an antigenome RNA of opposite polarity in order to synthesize more genomes [29], [30], [31]. The antigenome is typically less abundant than the genome since one antigenome template can be copied several times. In wildtype hosts, SINV and VSV produced a 6.3- and 5.5-fold excess of genomes over antigenomes, respectively (Fig. 2A). As expected for canonical siRNAs, the majority of VSV and SINV vsiRNAs were 21 nt in length (Fig. 2B,C). These mapped in roughly equal numbers to both genome and antigenome strands of SINV and VSV. Thus, the ratio of vsiRNAs derived from genome and antigenome strands was clearly different from the relative abundance of genomes and antigenomes (Fig. 2A–C). An equal distribution of vsiRNAs to both strands indicates that the preferred substrate for their biogenesis is sense-antisense viral dsRNA.

**Figure 3:**
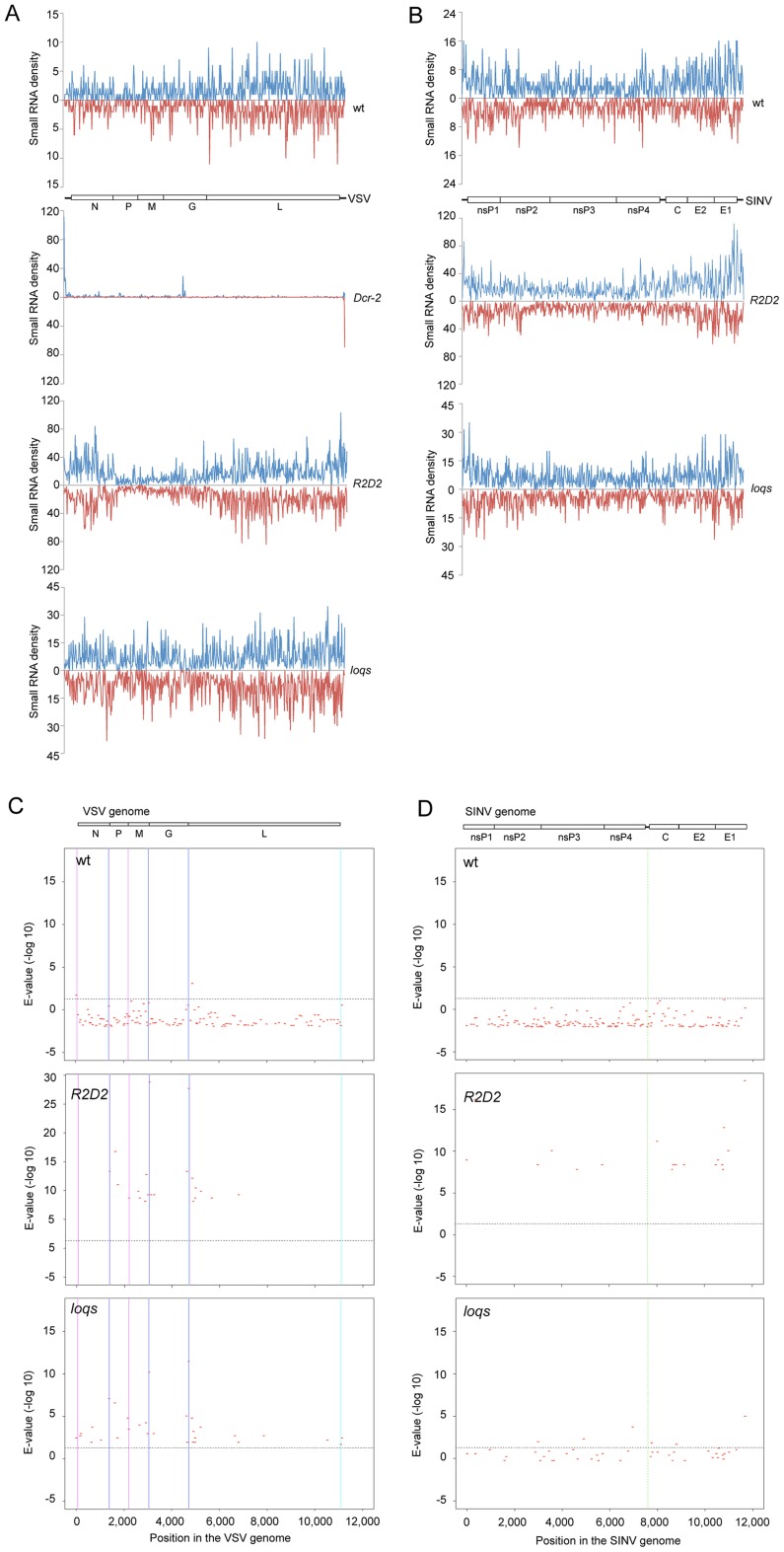
Characterization of vsiRNAs. (**A,B**) Coverage of vsiRNAs along viral genomes in samples from wildtype (wt), *Dcr-2*, *R2D2* and *loqs* mutant animals. Shown is read density in 20-nt bins for positive-stranded RNAs (blue) and negative-stranded RNAs (red) matching VSV (**A**) and SINV (**B**). Genome structures of the viruses are also shown oriented 5′ – 3′ for the positive strand. Protein-coding genes are highlighted. (**C,D**) Shown are the regions in the VSV (**C**) and SINV (**D**) genomes in which no vsiRNAs were detected by high-throughput sequencing. These gaps in vsiRNA coverage are scaled to the genome. Vertical lines in each plot mark the gene promoters within the VSV genome and the 5′ end of the subgenomic RNA in the SINV genome, respectively. The probability that each gap did not occur by chance is shown as the inverse expected value (E-value) on a log10 scale. The horizontal line in each plot represents a significance cutoff of *p* = 0.05 that the gap occurred by chance. E-values above the line are even more significant. Gaps are present in samples from wildtype (wt), *R2D2* and *loqs* mutant infected animals. Since there were fewer sequence reads in wildtype samples, the number of gaps are greater and their significance is smaller.

Processing of dsRNA by Dcr-2 is dependent on dsRNA substrate concentration in vitro [32], and thus substrate abundance is likely to affect the abundance of siRNAs *in vivo*. The ratio of siRNA product to dsRNA substrate is therefore an indirect measure of processing activity. Therefore, we normalized the levels of vsiRNAs to the levels of viral genomes (see Methods for details). We found that *Dcr-2* mutants had virtually no VSV vsiRNAs when compared to wildtype (Fig. 2B). This result confirmed that the 21 nt RNAs can be considered canonical vsiRNAs. In *R2D2* mutants, SINV vsiRNAs levels were similar to wildtype, and VSV vsiRNA abundance was slightly reduced (Fig. 2B,C). *loqs* mutants had little or no effect on the levels of VSV and SINV vsiRNAs. The distributions of vsiRNAs from *R2D2* and *loqs* mutants were homogeneous along the length of the viral genomes, as was also observed for wildtype (Fig. 3A,B). In contrast, vsiRNAs in *Dcr-2* mutants were strongly biased towards the 5′ ends of the VSV genome and antigenome (Fig. 3A). To summarize, R2D2 and Loqs-PD appear largely dispensable for Dcr-2-mediated biogenesis of VSV and SINV vsiRNAs.

**Figure 4:**
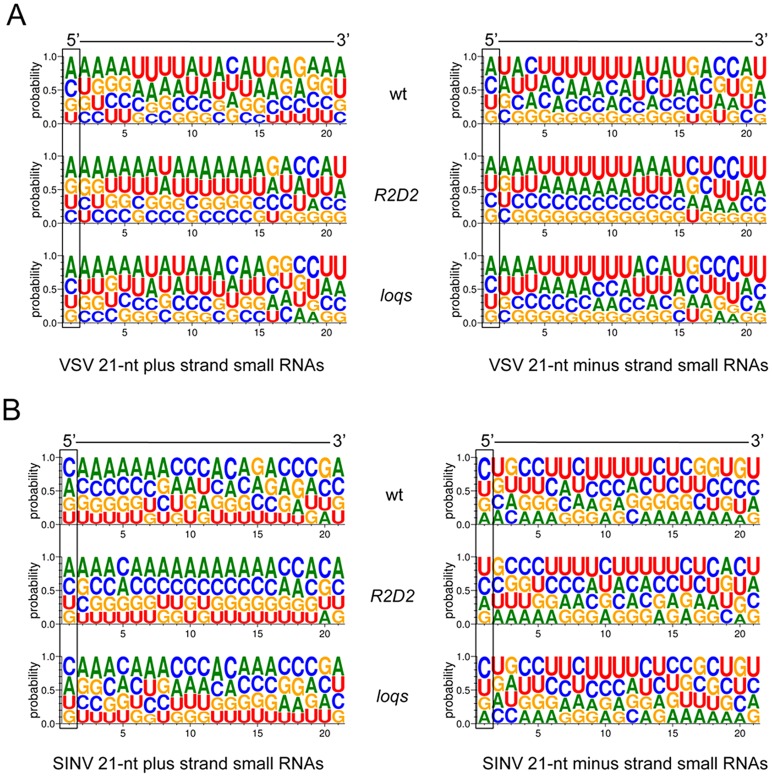
vsiRNAs show evidence of R2D2-dependent sorting. All vsiRNAs of the same strand from a sequenced library were pooled, and the frequency of base reads at each RNA position from 1 to 21 are shown in logo plots. The height of each base represents the relative frequency it was detected. Each RNA is aligned 5′ to 3′. Shown are vsiRNAs for VSV (**A**) and SINV (**B**) in samples from wildtype (wt), *R2D2* and *loqs* mutant animals. There is significant C-enrichment at the first position (*p*<0.05) in wildtype and *loqs* mutants but not in *R2D2* mutants. See Table S4 for detailed statistics.

R2D2 is essential for sorting and loading of exo- and endo-siRNAs onto Ago2. This can be detected *in vivo* by a characteristic enrichment of a C base, and sometimes, depletion of a U base at the 5′-end of loaded siRNAs [6], [33], [34], [35]. *R2D2* mutants exhibit loss of C enrichment at the 5′ end of endo-siRNAs [6], [36]. We asked whether R2D2 loads vsiRNAs onto Ago2 by looking for the nucleotide bias at the 5′ end of vsiRNAs. vsiRNAs derived from infection of wildtype animals showed significant C enrichment and U depletion at the 5′ end (Fig. 4A,B and Table S4). C enrichment was lost in *R2D2* mutants but was unaffected in *loqs* mutants. These results indicate that R2D2 has a sorting/loading function in the vsiRNA pathway that is similar to its role in the exo- and endo-siRNA pathway.

To determine if specific vsiRNAs are commonly made during infection, we calculated the pairwise correlation between different libraries. There was very low correlation for pairwise comparisons between wildtype, *loqs* and *R2D2* mutants (Table S5). However, low vsiRNA numbers made it difficult to make a definitive conclusion. Therefore, we compared our libraries to other libraries prepared from insects infected with SINV and VSV [22], [37]. The summary of this comparison is shown in Table S6. We found consistent results between all libraries in terms of the size, abundance, and coverage of vsiRNAs. However, there was also low correlation for pairwise comparisons between our libraries and those of Mueller et al [22] (Table S7). It is clear that library construction and sequencing platform can significantly influence the results of small RNA sequencing [38], [39], [40]. However, our inability to identify common individual vsiRNAs might be explained by heterogeneity of the viral dsRNA substrates subjected to Dcr-2 processing. Substrate heterogeneity is not unique to siRNAs. Sequencing of piRNAs in the *Drosophila* germline by different groups also failed to find common individual piRNAs despite reaching general conclusions about their origins and features [41], [42]. In contrast, identification of common individual miRNAs is possible when comparing different libraries due to the fact that miRNAs arise from well-defined precursors [6], [33].

### Mechanism of vsiRNA biogenesis

We sought to determine the source of sense-antisense viral dsRNA from which vsiRNAs were processed. Viral dsRNA can arise from virus transcription or genome-antigenome intermediates generated during replication. Although VSV and SINV generate similar types of replication intermediates, their transcription is very different. For VSV, the viral RNA polymerase transcribes its negative sense genome into a set of mRNA transcripts. Transcription initiates at the 3′ end of the viral genome, yielding an uncapped leader RNA of 47 nt, and then reinitiates at the nearby N gene promoter to produce capped and polyadenylated N mRNA [29], [31]. As it moves along the viral genome, the viral RNA polymerase reinitiates at internal promoters of the downstream genes and produces the corresponding capped and polyadenylated P, M, G, and L transcripts. Since some polymerase complexes fall off the template at intergenic junctions before reinitiating, the genes located near the 3′ end of the genome are expressed at higher levels than those located further downstream [31]. It was possible that VSV vsiRNAs were generated from transcript-genome hybrids or antigenome-genome duplexes. If they were generated from transcript-genome hybrids, then we predicted that intergenic promoter regions would be devoid of vsiRNAs. We examined the occupancy of vsiRNAs along the viral genome and detected several regions that exhibited no vsiRNA coverage (Fig. 3A,B). We then calculated the probability that each gap in coverage did not occur by chance (see Methods). Gaps with highly significant E-values in vsiRNA coverage included the regions between the N, M, G, P and L genes, close to or inside the intergenic promoters (Figs. 3C and S4). A low number of vsiRNA reads in the wildtype sample weakened our ability to detect significant gaps, though the E-values around gene promoters were more significant than the rest. However, highly significant gaps around gene promoters were consistently found in *R2D2* and *loqs* mutants. The gaps were detected in samples from *R2D2* mutants, which are competent for processing but not sorting of vsiRNAs. This suggests that the gaps are due to biases in processing. We also analyzed sequenced libraries of vsiRNAs prepared from VSV-infected DL-1 cells [43], S2 cells, and wildtype or *Ago2* mutant flies infected by VSV [22]. We observed highly significant vsiRNA gaps at gene promoters in these independent datasets, particularly the L promoter (Fig. S5). The simplest interpretation is that many vsiRNAs derived from central regions of the VSV genome are processed from genome-transcript hybrids. However, the absence of gaps at more distal gene promoters suggests that a significant fraction of VSV vsiRNAs from these regions come from genome-antigenome duplexes.

The sense SINV RNA genome also serves as a mRNA transcript for translation of viral proteins [30]. An additional subgenomic RNA is generated from the 3′ region of the SINV genome and functions as a mRNA transcript for structural proteins. Since there was no greater abundance of SINV vsiRNAs from the subgenomic region (Fig. 3B), it suggests that SINV vsiRNAs primarily derive from genome-antigenome duplexes. This has also been suggested by others [37]. We did not observe a reproducible pattern of significant gaps in vsiRNA coverage of the SINV genome, also consistent with the hypothesis that these vsiRNAs are generated from genome/antigenome duplexes (Fig. 3D).

**Figure 5:**
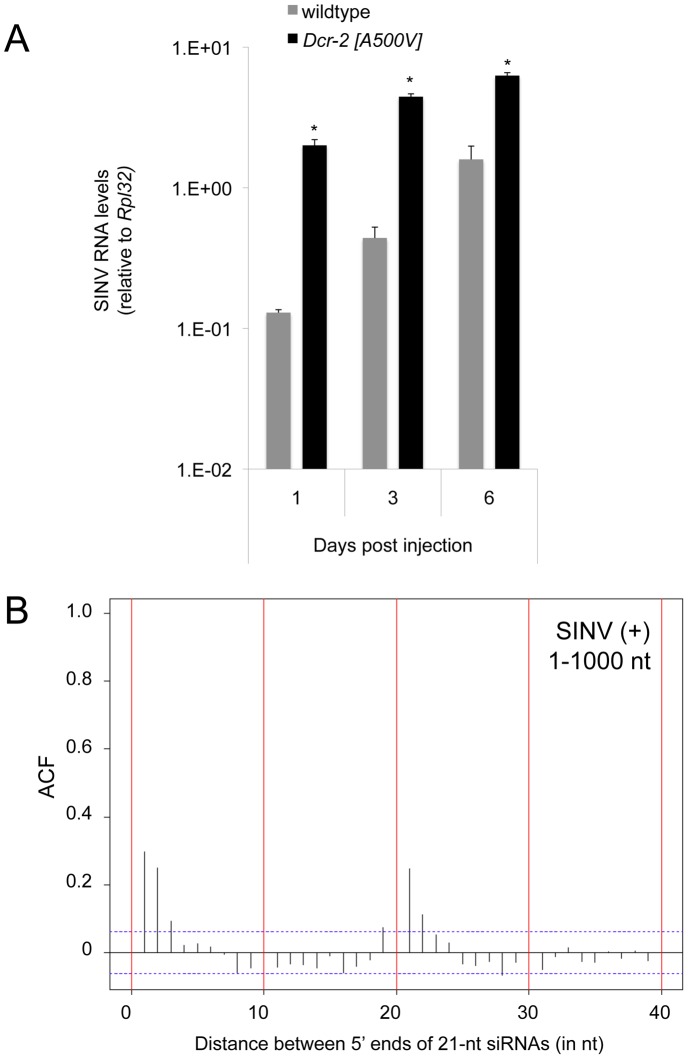
Requirement for Dcr-2 helicase activity and detection of vsiRNA phasing in SINV. (**A**) SINV genome RNA levels in *Dcr-2^A500V^* and wildtype animals at different times post infection. Asterisks indicate *p*<0.05. (**B**) Autocorrelation functions (ACF) of the distance in nucleotides between 5′ ends of vsiRNAs from the SINV positive strand. Shown are all vsiRNAs mapping to the 5′-most 1000 nts of the positive strand. The sample was derived from infected *R2D2* mutants. ACF values above the dotted line are statistically significant (*p*<0.05).

Our data indicates that vsiRNA production by Dcr-2 is an active mechanism that requires efficient processing of viral dsRNA substrates of diverse origins. Dcr-2 has RNase III domains that cleave dsRNA, and it also has an ATP-dependent helicase domain that is required for efficient processing [32]. To determine if the Dcr-2 helicase is essential, we infected a *Dcr-2* mutant that specifically disables the helicase domain (*Dcr-2^A500V^*) with SINV [44]. Similar to *Dcr-2* null mutants, *Dcr-2^A500V^* mutants showed increased levels of SINV replication (Fig. 5A). This result suggests that helicase activity is essential for the antiviral response. The helicase has been shown to enhance two features of dsRNA dicing. It is required for Dcr-2 to recognize dsRNA ends that are blunt or have 5′ overhangs [45]. It also allows multiple siRNAs to be produced along the length of a dsRNA without Dcr-2 dissociation [32]. One of the consequences of this Dcr-2 processivity is the production of siRNAs with defined spacing between the 5′ end of one siRNA and its nearest neighbors on the same strand [32]. This phasing has been detected in siRNAs generated from dsRNA substrates *in vitro* and *in vivo* as a discrete end-to-end distance peak [46], [47].

We wondered whether Dcr-2 processivity occurred along viral dsRNA substrates. Therefore, we examined the relationship of vsiRNAs to their neighbors as measured by end-to-end distance along the same strand (Fig. S6). We failed to detect phasing between VSV vsiRNAs (Fig. S6) and did not detect phasing in libraries prepared from other VSV-infected flies [22] (data not shown). A phasing signal was detected in VSV-derived siRNAs generated from cultured *Drosophila* DL-1 cells [43]. The reason for the differences between animal and cell culture studies remains unclear. For SINV, phasing was not detected in wildtype or *loqs* mutants (Fig. S6B). However, there was a phasing peak of 21 nts in *R2D2* mutants that was primarily due to vsiRNAs located within 1000 nts of the genome's ends (Fig. 5B and Fig. S6B). The stronger phasing signal near the genome ends suggests that processivity is weakened as Dcr-2 moves away from the genome ends. It further suggests that vsiRNA sorting by R2D2 is able to distort or mask the phasing signal. We also analyzed libraries prepared from SINV-infected mosquitoes [37] and cell lines [48], and observed a phasing peak from adult mosquitoes but none from cell lines (Fig. S7).

### Polyadenylated viral RNA is a preferential target of Ago2 slicing

**Figure 6:**
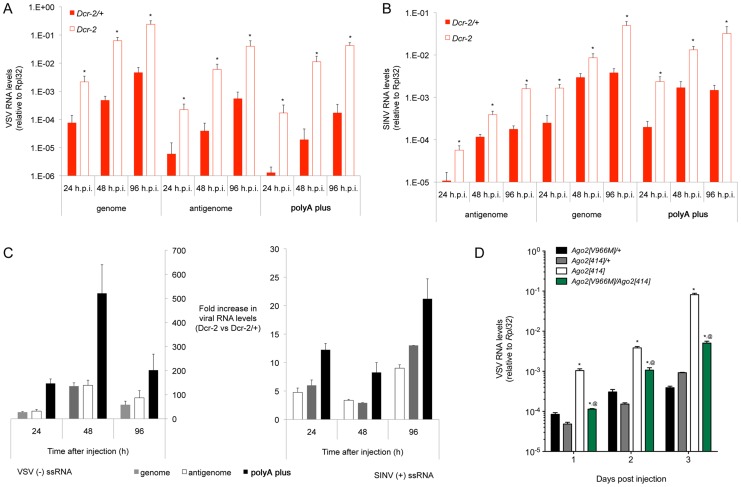
Viral polyadenylated RNA is a major target of slicing by Ago2. (**A,B**) Levels of genome RNA, antigenome RNA, and polyadenylated virus RNA from *Dcr-2* mutants (hollow bars) and wildtype controls (solid bars) at different times post infection with VSV (**A**) and SINV (**B**). Asterisks indicate *p*<0.05 comparing RNA levels between mutant and wildtype samples. (**C**) Fold increase in polyadenylated viral RNA, genome RNA, and antigenome RNA in *Dcr-2* mutants relative to wildtype at different times post infection with VSV or SINV. (**D**) VSV RNA levels in null *Ago2^414^*, *Ago2^V966M^/Ago2^414^*, and wildtype heterozygous animals at different days post infection. Asterisks indicate *p*<0.05 comparing RNA levels between mutant and matched wildtype samples; (@) indicates *p*<0.05 comparing RNA levels between *Ago2^414^* and *Ago2^V966M^/Ago2^414^* mutants.

vsiRNAs originate from both strands along the entire length of the VSV and SINV genomes, and so they could potentially inhibit positive-stranded, negative-stranded, and transcript viral RNAs. During infection, production of each viral RNA species is dependent on the others; genomes make transcripts; transcripts make replication proteins, which make genomes and antigenomes. Thus, vsiRNAs that directly inhibit one class of RNAs would indirectly inhibit production of other viral RNAs. We hypothesized that loss of inhibition would lead to more pronounced changes in the levels of direct vsiRNA targets than downstream RNAs. To measure the abundance of negative- and positive-stranded viral RNAs, we employed strand-specific RT-qPCR. We confirmed that mispriming did not significantly affect our measurements by using no-primer control reactions [49] (data not shown). We measured the abundance of polyadenylated viral RNAs by oligo dT-directed RT-qPCR. We then compared the abundance of viral RNAs extracted from wildtype hosts versus *Dcr-2* mutants. Levels of all SINV and VSV RNA species were derepressed in *Dcr-2* mutants (Fig. 6A,B). We calculated the level of derepression for each species of viral RNA. Polyadenylated viral RNA was more strongly derepressed than either negative- or positive-stranded viral RNA; 2.3-fold for SINV (*p* = 0.004) and 3.9-fold for VSV (*p* = 0.001) (Fig. 6C). This greater sensitivity of polyadenylated RNA to inhibition suggests that it is the primary target of vsiRNAs.

The SINV genome is of positive polarity and can also function as a transcript. However, not all SINV positive-stranded RNA is polyadenylated [50], explaining the difference we observed in repression of total positive-stranded SINV RNA versus polyadenylated SINV RNA. There were no significant differences in derepression of genome versus antigenome RNAs for either SINV (*p* = 0.49) or VSV (*p* = 0.48). If there was direct targeting of genomes and antigenomes, we predicted that the antigenome RNA levels would be more strongly affected. This is because vsiRNA levels from both strands are equivalent but the level of genome RNA greatly exceeds the level of antigenome RNA (Fig. 2).

R2D2 helps load siRNAs onto Ago2, and is also required to efficiently inhibit SINV and VSV replication. This would suggest that siRNA-loaded Ago2 (RISC) mediates the bulk of the inhibitory effect by slicing viral target RNAs. However, it is possible that cleavage of viral RNA by Dcr-2 is the major inhibitory mechanism, and Ago2 simply acts as a sink to drive the cleavage reaction. To distinguish between these mechanisms, we assayed an *Ago2* mutant with an amino acid substitution at position 966 (V→M) that impairs slicer activity of the protein [13]. We observed that mutant animals had significantly increased VSV RNA levels compared to controls (Fig. 6D). Since the point mutation impairs but does not completely ablate slicing activity, the effect on viral RNA silencing was not as great as observed with *Ago2* null mutants (Fig. 6D). These results indicate that the predominant inhibitory mechanism against VSV is mediated by Ago2 slicing activity.

## Discussion

Our results indicate the existence of a siRNA pathway dedicated to antiviral defense that is distinct from the one triggered by endogenous and exogenous dsRNA in *Drosophila*. The major difference between the two pathways seems to lie in the mechanism of siRNA biogenesis. In the antiviral pathway, virus dsRNA can be processed by Dcr-2 without Loqs-PD. In contrast, the canonical pathway relies upon Dcr-2 and Loqs-PD to process exogenous and endogenous dsRNAs. Downstream of processing, the two pathways appear to merge. vsiRNAs, exo-siRNAs, and endo-siRNAs are all sorted by a Dcr-2/R2D2 complex and loaded onto Ago2. These siRNA-Ago2 complexes inhibit target gene expression by a RNA slicing mechanism. Our results are consistent with other studies. Han et al [24] found that a weak *loqs* mutant had normal vsiRNA production and antiviral defense against FHV infection. However, it was possible that residual Loq-PD activity in the mutant rescued an antiviral function for the gene. We found that complete loss of Loqs-PD has no effect on antiviral silencing. Obbard et al [51] showed that *Ago2*, *R2D2* and *Dcr-2* are among the fastest evolving genes in the *Drosophila* genome. Since many host defense and pathogen genes co-evolve in a genetic arms race, rapid evolution of *Ago2*, *Dcr-2* and *R2D2* is possibly related to their antiviral functions [14], [52]. Strikingly, the *loqs* gene shows no sign of rapid evolution.

There are at least four possible interpretations of our results. First, Dcr-2 could process virus dsRNA in partnership with a dsRBP cofactor other than Loqs-PD. We have ruled out R2D2 as a potential substitute. Several other dsRBPs are encoded in the *Drosophila* genome, and two of these were found to interact with Dcr-2, but they are unlikely to mediate a global antiviral response since their expression is restricted to the male testis [53]. Moreover, no dsRBP gene other than *R2D2* has been identified as rapidly evolving as *Dcr-2* and *Ago2* [51]. A second explanation is that Dcr-2 alone processes virus dsRNA. *In vitro* studies have demonstrated that purified Dcr-2 protein efficiently processes dsRNA substrates and does not require a cofactor for its processing activity [32]. In fact, R2D2 inhibits the *in vitro* processing activity of Dcr-2 [32]. A third explanation is that the Dcr-2/Loqs-PD heterodimer recognizes and processes virus dsRNA, but unlike other substrates, the presence of Loqs-PD is not essential. Note that the molecular function of Loqs-PD in Dcr-2 processing activity *in vivo* is still unknown. A fourth explanation is that the Dcr-2/R2D2 heterodimer recognizes and processes virus dsRNA, although processing is not affected by the absence of R2D2. If virus dsRNA is processed by Dcr-2/R2D2, then vsiRNA products could be directly loaded onto Ago2 and avoid loading competition with endogenous siRNAs. This might enhance the antiviral response.

If Dcr-2 acts on virus RNA without the need of a dsRBP cofactor, then how does the enzyme recognize virus RNA as different from other types of dsRNA? Purified SINV RNA injected into cells replicates over time in a manner that is unaffected by Loqs-PD. Thus, it is not virion structure or mode of entry that signals Dcr-2 to differentially recognize virus RNA. Instead, it indicates that Dcr-2 specifically recognizes something intrinsic to the virus RNA or its intermediates. Preliminary experiments injecting *in vitro* synthesized SINV RNA into cells also show no effect of the *loqs* mutant on RNA replication (data not shown). Therefore, it is unlikely that Dcr-2 recognizes chemical modifications of SINV RNA as the distinguishing feature. If Dcr-2 does not recognize modified features of virus RNA, what is the nature of the signal? RNA virus transcription and replication are typically sequestered into ribonucleoprotein “factories” that contain concentrated levels of RNA and enzymes [29], [30], [54]. This is distinct from exo- and endo-dsRNAs, which can be found dispersed within a cell. Limited accessibility of viral dsRNA by Dcr-2/Loqs-PD could be one reason that dsRNA processing is indifferent to these complexes. Alternatively, greater substrate heterogeneity might distinguish virus dsRNA from other kinds of dsRNA. In this regard, we have found the Dcr-2 helicase domain is required for antiviral silencing and, at least *in vitro*, is also necessary for Dcr-2 to recognize non-canonical ends of dsRNA duplexes [45].

Our work also addresses the origin of vsiRNAs. Others have suggested that viral replication intermediates are the exclusive substrates for vsiRNA production [20], [21], [22], [24]. Our analysis of SINV is consistent with a replication intermediate exclusive mechanism. However, we find evidence that both replication intermediates and transcript-genome hybrids can be precursors for VSV vsiRNAs. Our analysis also has explored how Dcr-2 cleaves the virus dsRNAs. When Dcr-2 processively cleaves dsRNA, initiating from ends that are common to different dsRNA molecules, a phasing signal is seen in sequence data. No phasing is seen if Dcr-2 is not processive or if dsRNA ends are highly heterogeneous. For SINV, there is a weak sign of phasing. Indeed, we detect stronger phasing of SINV vsiRNAs near the ends of the genome, where SINV dsRNAs would tend to have common ends.

What is the mechanism by which vsiRNAs inhibit viral replication? Some have proposed that Dcr-2 mediated processing of viral dsRNA is primarily responsible for the reduction seen in viral RNA levels [20]. Alternatively, vsiRNAs loaded onto Ago2 could potentially carry out many rounds of virus RNA destruction because RISC is a multiple turnover enzyme [55]. Two lines of evidence indicate it is the latter mechanism that mediates the bulk of VSV inhibition. First, R2D2 shows an antiviral activity that is comparable to the antiviral activity of Dcr-2 (Fig. 1). Since R2D2 sorts and loads vsiRNAs downstream of Dcr-2 mediated processing, it suggests that loading of Ago2 is required for the mechanism. Second, we show that Ago2 slicer activity is required for silencing of VSV RNA. Thus, Ago2 is not merely acting to sequester free vsiRNAs in order to drive the dsRNA processing reaction. Rather, vsiRNA-loaded Ago2 slices viral RNAs and substantially contributes to the inhibitory mechanism.

## Materials and Methods

### *Drosophila* stocks, viruses and reagents

All mutant alleles used in this study were previously described. The different *Drosophila* mutants analyzed were: *Dcr-2^L811fsX^* and *Dcr-2^A500V^* [44], *R2D2^1^/R2D2^S165fsX^* [18], [56], *loqs* ^f00791^/*loqs* ^KO^ [10], [57] and *loqs* ^KO^ *R2D2* ^S165fsX^/*loqs* ^f00791^ *R2D2* ^1^ [6], *Ago2* ^414^ [58], *Ago2* ^V966M^ [13] and *loqs* ^KO^ PB and PD rescue lines [8]. Wildtype referred to in this study had each mutation in trans to wildtype chromosomes, making a heterozygous state. Chromosome 2 had an FRT42D insertion. All stocks tested negative for the endosymbiont *Wolbacchia*, which has been shown to influence *Drosophila* antiviral defense [59]. SINV, SINV-GFP and VSV-GFP were a kind gift from Dennis Brown, Ilya Frolov and Curt Horvath, respectively. Viruses stocks were prepared and titered in BHK-21 cells as described previously [25], [60]. The titers for VSV and SINV used in this study were 5×10^8^ pfu/mL and 4×10^10^ pfu/mL, respectively.

### Infections

To avoid possible complications related to differences in background, microbiota or rearing, we crossed heterozygous animals bearing mutant alleles to each other, and we infected their mutant and heterozygous wildtype offspring at the same time. These then served as mutant and wildtype control samples for each experiment. We utilized a microinjector to inject 50 nl of a PBS solution containing the viruses into the thorax of 2–4 day old female adults. Animals were injected with 5,000 pfu of VSV and 20,000 pfu of SINV in all experiments.

### Survival analysis

For the survival analysis, three groups of 20 adults of each genotype were injected separately and survival was monitored daily. Each experiment was repeated at least three times. Survival graphs and median survival were plotted and calculated using Prism (GraphPad). A two-tailed student *t* test was used to statistically analyze differences in median survival between groups.

### Analysis of viral RNA replication by quantitative PCR

Total RNA from adults was extracted using Trizol reagent according to the manufacturer's protocol (Invitrogen). 1 µg of total RNA was reverse transcribed using 250 ng of random primers, 500 ng of anchored oligo dT primers or 2 pmol of gene and strand specific primers per reaction. The resulting cDNA was used as template for qPCR reaction containing Sybr Green (Invitrogen) and primers specific for the amplification of the genes of interest. The relative amount of the indicated RNAs normalized to an internal control (GAPDH, Rpl32 or Actin 5C) was calculated using the delta Ct method. A two-tailed student *t* test was used to statistically analyze differences in viral RNA transcript levels between control and mutant animals. For strand specific qPCR, 2 pmols of primers for one strand were used during reverse transcription. Reverse transcription reactions were performed in the absence of primers or enzyme as negative controls for qPCR to ensure the identity of the products. Oligonucleotides designed in this study for RT and qPCR are described in Table S8. *Rpl32*, *GFP* and *Actin5* were used as normalization standards as described previously [6].

### SINV RNA extraction and embryo injections

Embryo injections were performed as described [6]. Genomic SINV RNA was extracted from purified virions using Trizol (Invitrogen). The RNA was diluted to a concentration of 200 ng/µl in 0.1 mM NaPO_4_ pH 7.8, 5 mM KCl solution for the injections. Female and male adults of a given genotype were placed inside an egg collecting cage, and eggs were collected every hour at 25°C, dechorionated, and injected within the next 45 minutes. SINV RNA was injected at the posterior end of eggs with a volume of ∼100 pL. After injection, embryos were incubated at 23°C under halocarbon oil in an oxygenated chamber, and harvested after 2 or 24 hours post injection. They were directly put into 100 µl of Trizol for RNA extraction. An average of 30 embryos were pooled per sample per time point. RNA was reverse transcribed using anchored oligo-dT primers, and qPCR reactions were performed with SINV-specific primers at the 3′ end of the SINV sense genome (Table S3). A two-tailed student *t* test was used to statistically analyze differences in viral RNA transcript levels. The experiment was repeated four times with similar results. The endogenous gene *Rpl32* qPCR was used as normalization standard.

### Preparation of small RNA libraries, deep sequencing and data analysis

For the construction of the small RNA libraries, total RNA was isolated from adults at 48 h post injection of virus using Trizol (Invitrogen). Low molecular weight (LMW) RNA was prepared from total RNA, and small RNAs between the 18–34 nt size range were PAGE purified from the LMW RNA as described previously [6]. 200 ng of the small RNA preparation was used to prepare a library using the SOLiD total RNA expression kit according to the manufacturer's protocol (Ambion). Sequencing of the libraries was performed using the SOLiD platform according to the manufacturer's protocol (ABI) at the Genomics Core of the Feinberg School of Medicine (Northwestern University). Sequencing reads were aligned to release 5.2 of the reference *Drosophila* genome, VSV (J02428.1) and SINV (J02363.1) genomes deposited on NCBI using the Small RNA Analysis Pipeline Tool (Rna2Map) available from Applied Biosystems. Briefly, Rna2Map uses the Mapreads program from Applied Biosystems to simultaneously align reads to a reference and to filter out contamination from sequencing adaptors. Mapping was done allowing up to one mismatch in color space for the overall alignment. Reads aligning to the *Drosophila* and viral genomes were retained, whereas reads aligning to sequencing primers were removed from further analysis.

Reads mapping to the *Drosophila* reference genome were further analyzed as described previously [6]. Briefly, reads mapping to miRNAs were found by collecting the coordinates of known *Drosophila* miRNAs from Flybase v5.18 and searching the genomic alignment for reads overlapping these coordinates. The same process was repeated for mRNAs and transposons, where mRNA coordinates were taken from Flybase and transposons coordinates were taken from version dm3 of the UCSC RepeatMasker annotations. Reads mapping to rRNA sequences were determined by filtering transposon, miRNA, and mRNA reads from the *Drosophila* genome alignment and matching the remaining reads against rRNA sequences from Flybase v5.18. Ad hoc perl scripts were used in all steps, including the calculation of read size/strand distributions. The numbers of reads (>16 nt) mapping to rRNA, mRNA, miRNA and TEs for each sample are detailed in Tables S2 and S3. The total number of reads aligning to the *Drosophila* genome was used to normalize the libraries to allow for comparison between different libraries. Abundance of specific *Drosophila* small RNAs was plotted as the number of reads in a thousand reads from the total number of reads each library described [48]. Although minor distortions have to be taken into account, we believe the major conclusions of our analysis are not affected by this normalization.

Reads mapping to the VSV and SINV genomes were first normalized to the total size of the library as described above. Viral genome RNA levels were then used to normalize vsiRNA numbers to allow comparison between the different experimental samples. Importantly, viral genome RNA levels were determined by strand specific PCR in the total RNA extracted from the same animals that were used to make the small RNA libraries.

The sequencing datasets were deposited on the Gene Expression Omnibus website at the NIH. Accession numbers are: GSE36449 GSM893954 GSM893955 GSM893956 GSM893957 GSM893958 GSM893959 GSM893960.

For all the subsequent analyses (weblogo, phasing, occupancy and gaps), we separated only 21-nt reads mapped against the virus reference.

### Analysis pipeline for the comparison between SOLiD and Illumina libraries

In order to compare our sequencing results to the results of other groups using different platforms [22], [37], we created a different analysis pipeline that could be applied to all strategies. We did this to avoid any potential differences that could be caused by the bioinformatic analysis and not the library construction strategy and sequencing platform used. The sequencing datasets were obtained from the SRR database at the NCBI website under accession numbers SRR059800, SRR059801 and SRR059803 from Mueller et al [22] and SRR400496 from Myles et al [37]. The summary of these results are on Table S6. Briefly, the libraries were analyzed through an automated pipeline containing three main steps. In the first step, reads from SOLiD were converted from color space to base space and filtered using scripts from Solid Software Tools (http://www.appliedbiosystems.com/absite/us/en/home/applications-technologies/solid-next-generation-sequencing/ngs-data-analysis-software/software-community.printable.html).

Reads from Illumina were filtered using fastx-toolkit (http://hannonlab.cshl.edu/fastx_toolkit/index.html). In both cases, reads below the minimum quality threshold were discarded. In the second step, adapters were removed using the cutadapt software (http://code.google.com/p/cutadapt/). In the third step, the remaining reads were mapped against the virus genome references using SHRiMP [61] considering only single best mapping and a minimum of 80% similarity. For all the subsequent analyses (weblogo, phasing, occupancy and gaps), we separated only 21-nt reads mapped against the virus reference.

### Weblogo analysis

Nucleotide probability cartoons for small RNAs were generated using Weblogo 3 (http://weblogo.threeplusone.com/create.cgi).

For each sample, we tested whether the base C is enriched at the first position of 21-nt reads compared to two different references: the genome-wide base composition and, separately, compared to the base composition from all reads used to make the weblogos. Similarly, we also tested whether the base U is depleted at the first position. For example, the genome-wide base composition for SINV is 0.283, 0.261, 0.249 and 0.208 for A/C/G/U respectively. Thus for testing the enrichment of the base C, we are testing the hypotheses as follows:
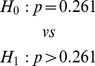



For testing the depletion of U, the hypotheses are:
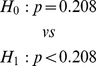



The p-values were calculated based on the Z-test for the one-sample proportion.

### Phasing analysis

Let *X_i_* and *Y_i_* be the frequency of observed sequence reads on the sense and antisense strands starting at position *i* for *i* = 1,…,*n* where *n* is the length of the entire genomic region. We use the standard auto-correlation function (ACF) from R software to investigate whether the read starting positions from the same strand are correlated. The 95% cutoff line for positive correlation or negative correlation (the null hypothesis is that the correlation = 0 at each lag) is shown in the plots. If the auto correlation at a given lag exceeds the cutoff line it can be regarded as significant (either >0 or <0, depending on which direction the correlation goes).

### Significance test for the occupancy gaps

We defined the occupancy of any given position as the total number of reads (sense+antisense) that cover this position. A gap is defined as a region that is not covered by any reads from the sense or antisense strand. Suppose we observe a gap of length *k* from position *j* to *j*+*k*−1 (sense strand position). This implies that there are no reads starting at position *j*−20 to *j*+*k*−1 on the sense strand, and no reads starting at positions *j* to *j*+*k*−1+20 on the antisense strand. Suppose we observe a total of *T*_1_ and *T*_2_ reads from the sense and antisense strands respectively and the length of entire region is *n*. Under the null hypothesis, that is, the reads are evenly distributed across the entire region and the reads distribution on the two strands are independent, then we can use a Poisson distribution with mean *T*_1_/*n* and *T*_2_/*n* respectively to approximate the reads count distribution at each position given *n* is very large. Let *p*_1_(0) and *p*_2_(0) be the zero-probabilities under the two Poisson distributions. The probability that we observe a gap of exact length *k* is given by *p*(*k*) = *p*_1_ *^k^* ^+20^(0)*p*_2_ *^k^* ^+20^(0)(1−*p*_1_(0) *p*_2_(0))^2^ (i.e. a gap of exact length *k* requires co-occurrence of no reads starting in the *k*+20 bp range in either strand, and in addition that in the immediate upstream or downstream base pair of the two stands cannot simultaneously both have gaps). The p-value of a gap of length *k*, defined as the probability to observed a gap of length *k* or even longer is given by *p_value* = ∑*_m_*_≥*k*_ *p*(*m*) for integer *m*. *m* is the summation index, i.e. sum over integer *m*≥*k*+20. The expected value (E-value) thus is approximately *n*×*p_value* where *n* is the length of the entire genomic region.

### Pairwise tests

To test whether two conditions have different distribution patterns of reads count along the entire region for wildtype and mutant samples, we first divided each mRNA transcript into bins. Due to the very small sample size, when we comparing widltype to either *R2D2* or *loqs* mutants, we used a relatively larger bin size of 50 nt. For comparing the two mutants we used a bin size of 20 nt instead. The entire region contains five different transcripts with the start and end positions as follows: start = (51,1386,2209,3049,4723) end = (1376,2199,3039,4713,11095). We tested the difference for each bin on each strand within each transcript sequentially. For a given transcript, suppose we observe a total of *T*_1_ and *T*_2_ tags on one strand for the two conditions respectively. For a particular bin under testing, let *X* and *Y* be the number of reads that start in this bin. We consider a Poisson distribution to approximate the sampling distribution of *X* and *Y*, the mean parameters of which are denoted as *λ*_1_ and *λ*_2_. The null hypothesis is, the reads distribution pattern everywhere is the same between these two conditions. Therefore the mean parameters *λ*_1_ and *λ*_2_ are proportional to the total number of reads observed in the two conditions. Given the observed total number of reads, the null hypothesis can be stated as:
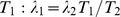



We can test this hypothesis based on the conditional distribution of *X*|(*X*+*Y*), which is known as a binomial distribution:
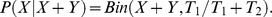



A two-sided *p*-value is calculated based on this conditional distribution for each bin. In the plot, we plotted the log10 of the *p*-value with a “+” or “−” sign attached as follows. If *P(X*≥*x*|*X*+*Y*)≤0.5, which indicates the observed *x* is in the right tail, we attach a “+” sign, and otherwise a “−” sign. A “+” sign essentially means condition 1 has more reads than expected under the null, and “−” sign means the opposite.

## Supporting Information

Figure S1 **Survival of animals after infection.** Survival of heterozygous wildtype, *R2D2*, *loqs*, and *loqs R2D2* mutant animals after treatment. Animals were untreated (solid circles), mock injected (solid squares), SINV injected (hollow triangles), and VSV injected (hollow squares). The means and standard deviations for at least three independent experiments are shown.
(TIF)

Figure S2 **The distribution of small RNAs matching the** ***Drosophila*** **genome is not affected by** ***R2D2*** **and** ***Loqs*** **.** The binned numbers of sequenced small RNAs that map to the second and third chromosomes of wildtype (wt), *R2D2* and *loqs* mutants infected with VSV (**A**) or SINV (**B**). The number of reads is in log10 scale for reads on the positive strand (red) and negative strand (orange).
(TIF)

Figure S3 **21-nt small RNAs derived from the** ***Drosophila*** **genome are dependent on Dcr-2, R2D2 and Loqs.** Frequency distribution of small RNAs derived from the *Drosophila* genome displayed by RNA length. Shown are samples prepared from wildtype (wt), *Dcr-2*, *R2D2* and *loqs* mutants infected with VSV (**A**) or SINV (**B**).
(TIF)

Figure S4 **Transcription promoters of VSV showing gaps in vsiRNA coverage.** Shown are the regions in the VSV genome that surround the promoters for the P, M, G, and L genes. Non-transcribed promoters are defined by the blue and pink vertical lines in each plot. Also displayed are regions in which no vsiRNAs were detected by high-throughput sequencing. These gaps in vsiRNA coverage are scaled to the genome. The probability that each gap did not occur by chance is shown as the inverse expected value (E-value) on a log10 scale. The horizontal line in each plot represents a significance cutoff of *p* = 0.05 that the gap occurred by chance. E-values above the line are even more significant. Gaps are present in samples from *R2D2* (**A**) and *loqs* (**B**) mutant infected animals.
(TIF)

Figure S5 **Analysis of gaps in vsiRNA coverage over the VSV genome as detected by independent sequencing experiments.** Shown are the regions in the VSV genome in which no vsiRNAs were detected by high-throughput sequencing performed by Mueller et al [22] (S2 cells, wildtype (wt), and *Ago2* mutants) and Sabin et al. [43] (DL-1 cells). These gaps in vsiRNA coverage are scaled to the genome. Vertical lines in each plot mark the gene promoters within the VSV genome. The probability that each gap did not occur by chance is shown as the inverse expected value (E-value) on a log10 scale. The horizontal line in each plot represents a significance cutoff of *p* = 0.05 that the gap occurred by chance. E-values above the line are even more significant. Note that the L gene promoter most consistently shows significant gaps in vsiRNA coverage.
(TIF)

Figure S6 **Phasing analysis of vsiRNAs derived from the positive and negative strands of SINV and VSV.** Autocorrelation functions (ACF) of the distance in nucleotides between 5′ ends of vsiRNAs from VSV (**A**) and SINV (**B**) positive (+) and negative (−) strands, as indicated. The samples were generated from infected wildtype (wt), *R2D2*, and *loqs* mutants. ACF values above the dotted line are statistically significant (*p*<0.05).
(TIF)

Figure S7 **Phasing analysis of vsiRNAs derived from the SINV genome after infection of mosquitoes and** ***Drosophila*** **.** Autocorrelation functions (ACF) of the distance in nucleotides between 5′ ends of vsiRNAs from SINV positive (+) and negative (−) strands. Shown are all vsiRNAs mapping to the 5′-most 1000 nts of the relevant strand. Samples were from our *R2D2* mutant *Drosophila* (**A,B**), mosquitoes from Myles et al [40] (**C,D**), the cell line Aag2 (**E,F**), and cell line U4.4 (**G,H**) from Vodovar et al [48]. ACF values above the dotted line are statistically significant (*p*<0.05).
(TIF)

Table S1 **Statistical analysis of the differences in median survival with viral infection of different** ***Drosophila*** **mutants.**
(PDF)

Table S2 **Summary of the raw data from the sequencing of the small RNA libraries described.**
(PDF)

Table S3 **Percentage of reads in each feature class for the sequenced libraries.**
(PDF)

Table S4 **Statistical analysis of C-enrichment and U-depletion at the first position of 21-nt vsiRNA sequence reads**
(PDF)

Table S5 **Pairwise correlation of vsiRNA density along viral genomes**
(PDF)

Table S6 **Comparative analysis of the SOLiD and Illumina libraries from this work and other published studies.**
(PDF)

Table S7 **Pairwise correlation of vsiRNA density along the VSV genome between the libraries in this work and Mueller et al.** [**22**] **.**
(PDF)

Table S8 **Oligonucleotides used in experimental analysis.**
(PDF)
