# Correction: Functional Specialization of the Small Interfering RNA Pathway in Response to Virus Infection

**DOI:** 10.1371/annotation/4e52dfe0-479d-4be7-8545-b4ee8a1eb9ed

**Published:** 2013-10-22

**Authors:** Joao Trindade Marques, Ji-Ping Wang, Xiaohong Wang, Karla Pollyanna Vieira de Oliveira, Catherine Gao, Eric Roberto Guimaraes Rocha Aguiar, Nadereh Jafari, Richard W. Carthew

The following information was missing from the Funding section: "JTM, KPVO and ERGRA received funding from CAPES, CNPq and FAPEMIG."

