# Supplementary figures and images for: Functional Specialization of the Small Interfering RNA Pathway in Response to Virus Infection
Source: PLoS Pathog. 2013 Aug 29;9(8):e1003579. doi: 10.1371/journal.ppat.1003579 (PMC3757037; doi:10.1371/journal.ppat.1003579)

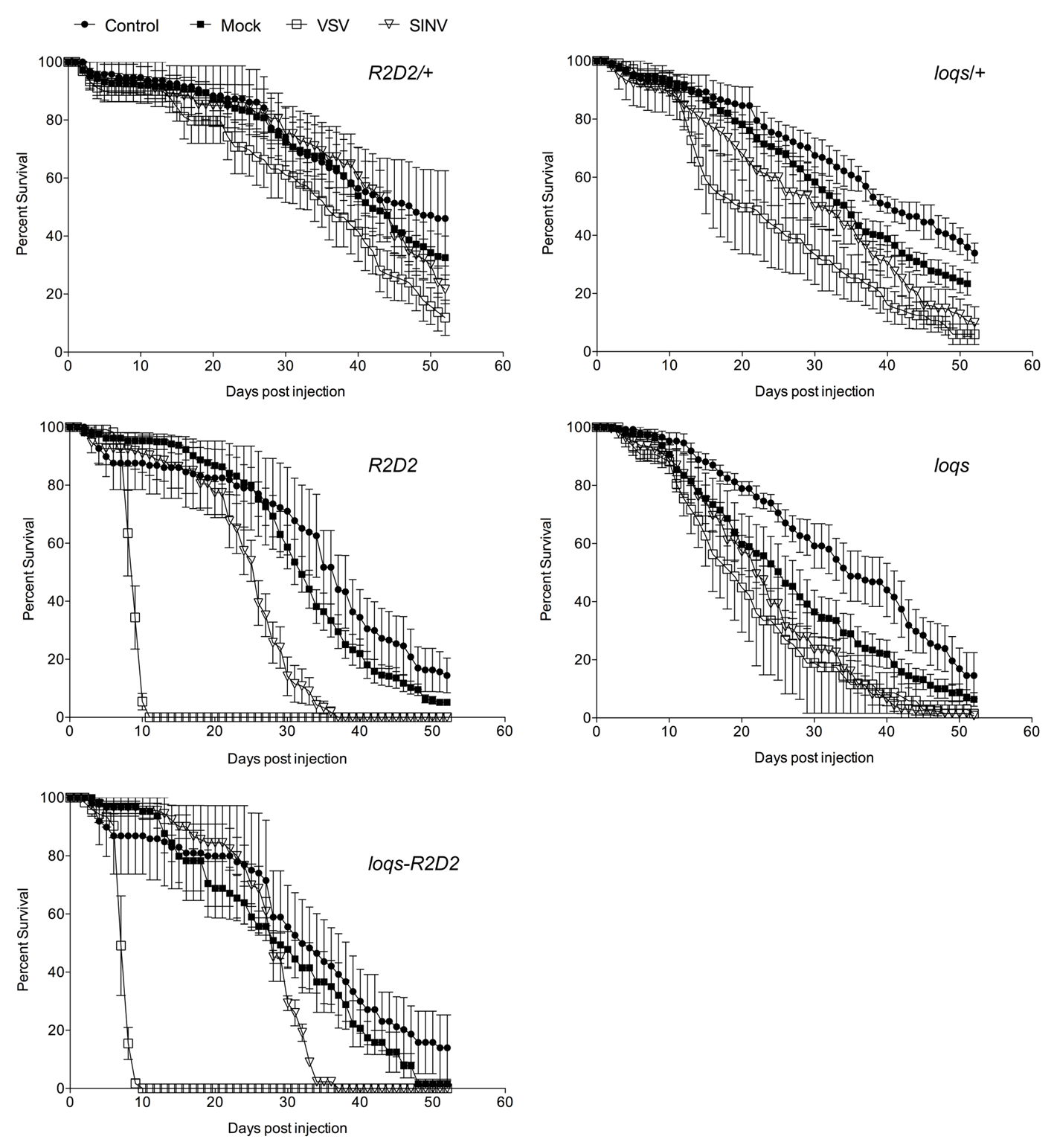

Supplement: Figure S1 — Survival of animals after infection. Survival of heterozygous wildtype, R2D2, loqs, and loqs R2D2 mutant animals after treatment. Animals were untreated (solid circles), mock injected (solid squares), SINV injected (hollow triangles), and VSV injected (hollow squares). The means and standard deviations for at least three independent experiments are shown. (TIF) [file ppat.1003579.s001.tif]

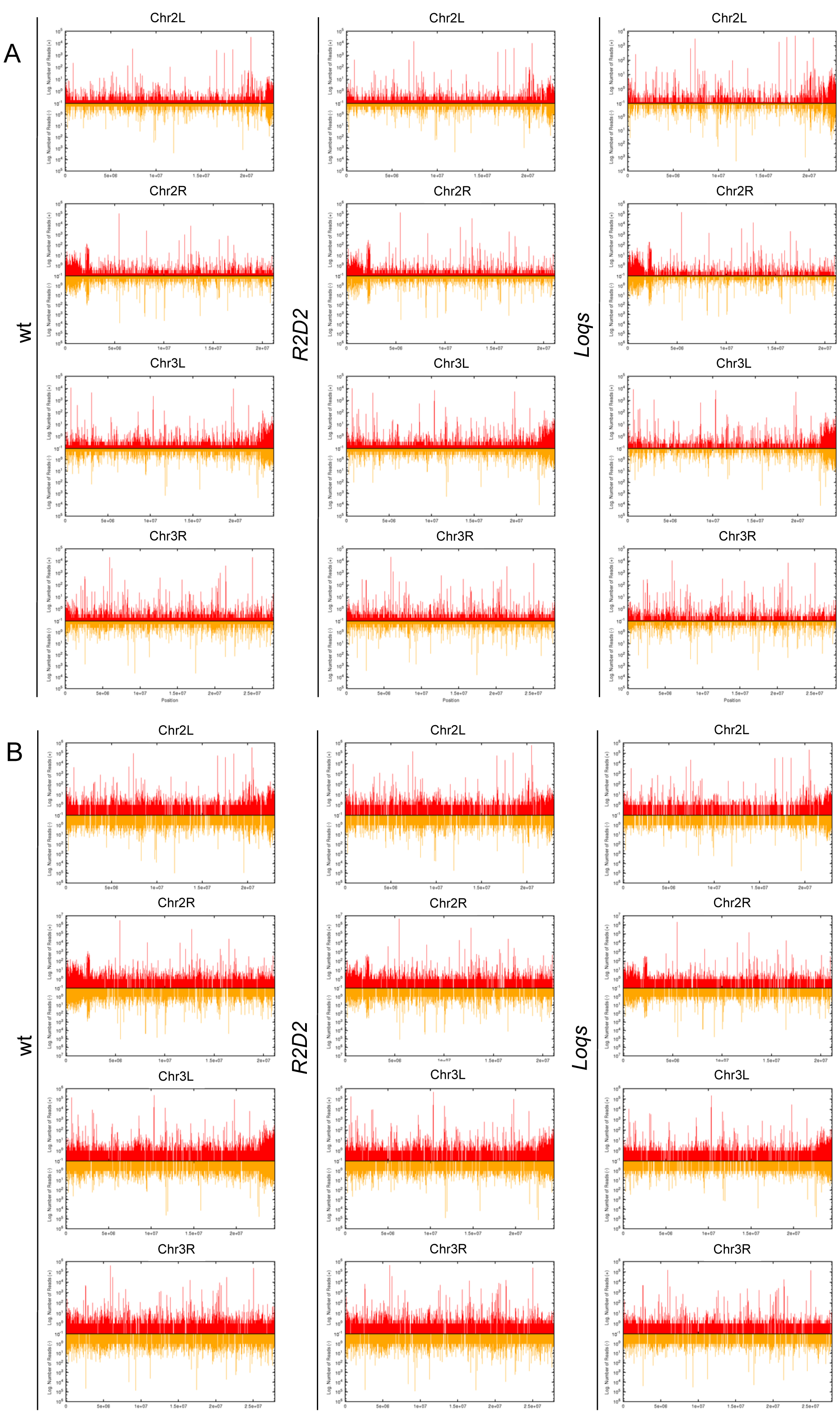

Supplement: Figure S2 — The distribution of small RNAs matching the Drosophila genome is not affected by R2D2 and Loqs . The binned numbers of sequenced small RNAs that map to the second and third chromosomes of wildtype (wt), R2D2 and loqs mutants infected with VSV (A) or SINV (B). The number of reads is in log10 scale for reads on the positive strand (red) and negative strand (orange). (TIF) [file ppat.1003579.s002.tif]

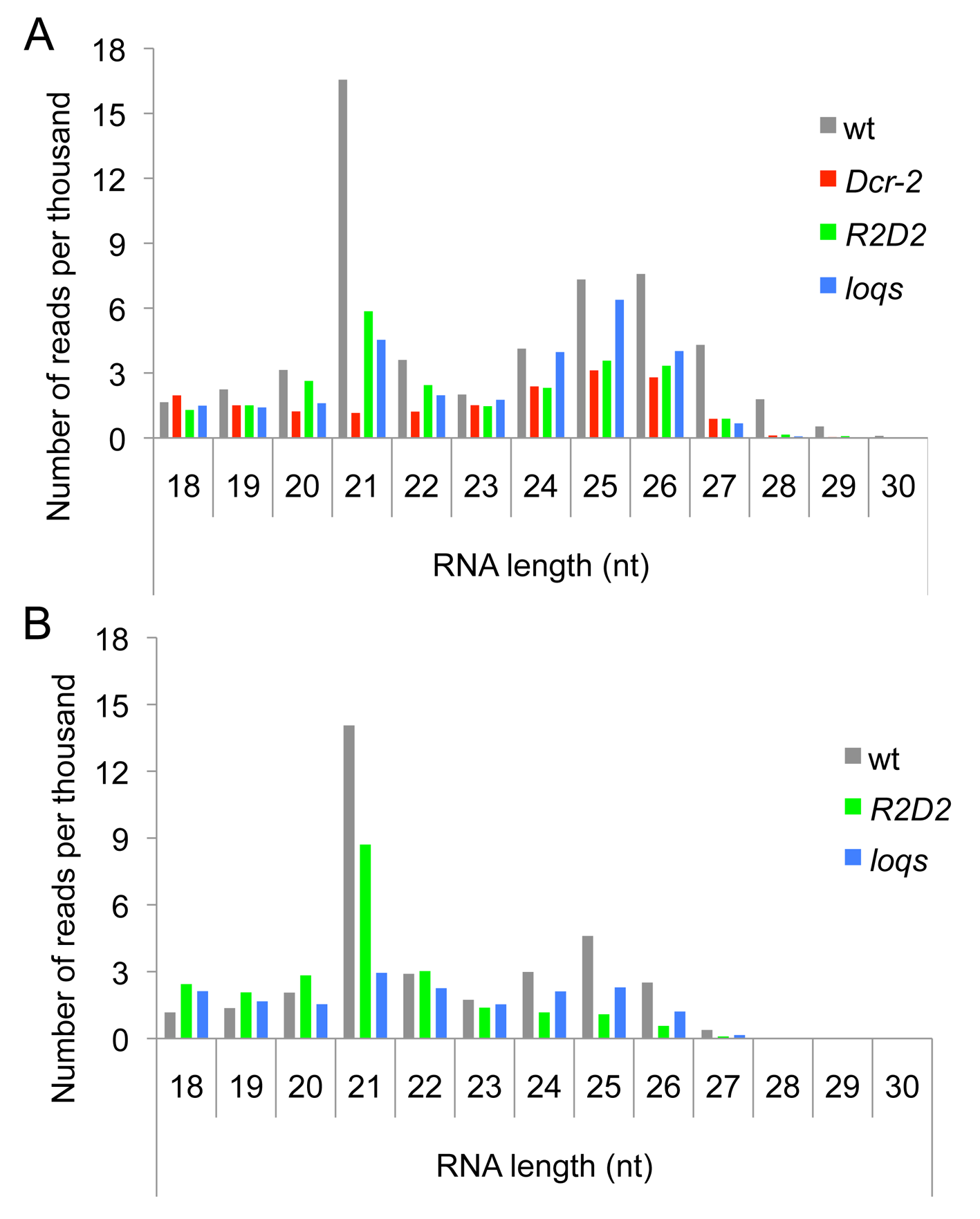

Supplement: Figure S3 — 21-nt small RNAs derived from the Drosophila genome are dependent on Dcr-2, R2D2 and Loqs. Frequency distribution of small RNAs derived from the Drosophila genome displayed by RNA length. Shown are samples prepared from wildtype (wt), Dcr-2, R2D2 and loqs mutants infected with VSV (A) or SINV (B). (TIF) [file ppat.1003579.s003.tif]

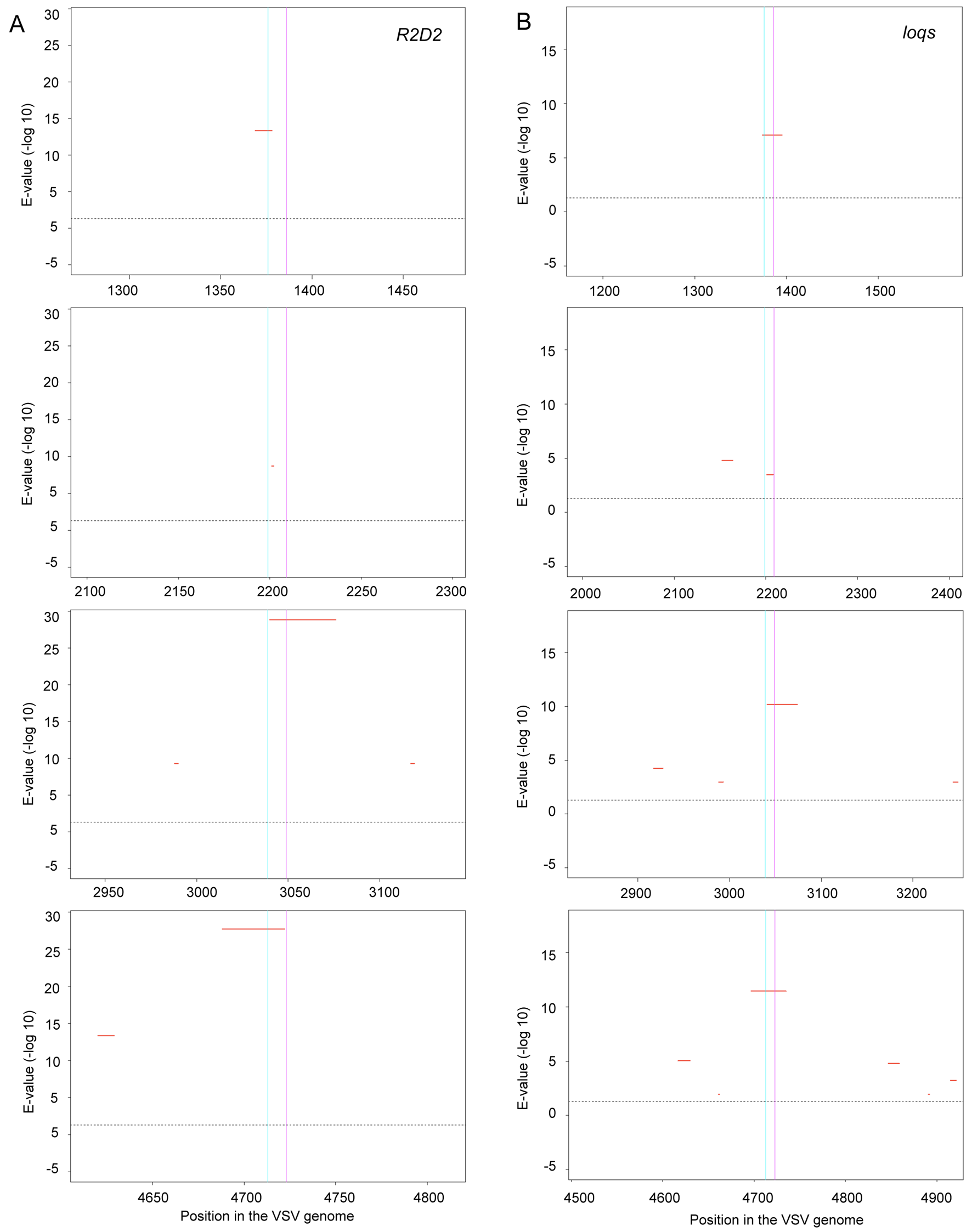

Supplement: Figure S4 — Transcription promoters of VSV showing gaps in vsiRNA coverage. Shown are the regions in the VSV genome that surround the promoters for the P, M, G, and L genes. Non-transcribed promoters are defined by the blue and pink vertical lines in each plot. Also displayed are regions in which no vsiRNAs were detected by high-throughput sequencing. These gaps in vsiRNA coverage are scaled to the genome. The probability that each gap did not occur by chance is shown as the inverse expected value (E-value) on a log10 scale. The horizontal line in each plot represents a significance cutoff of p = 0.05 that the gap occurred by chance. E-values above the line are even more significant. Gaps are present in samples from R2D2 (A) and loqs (B) mutant infected animals. (TIF) [file ppat.1003579.s004.tif]

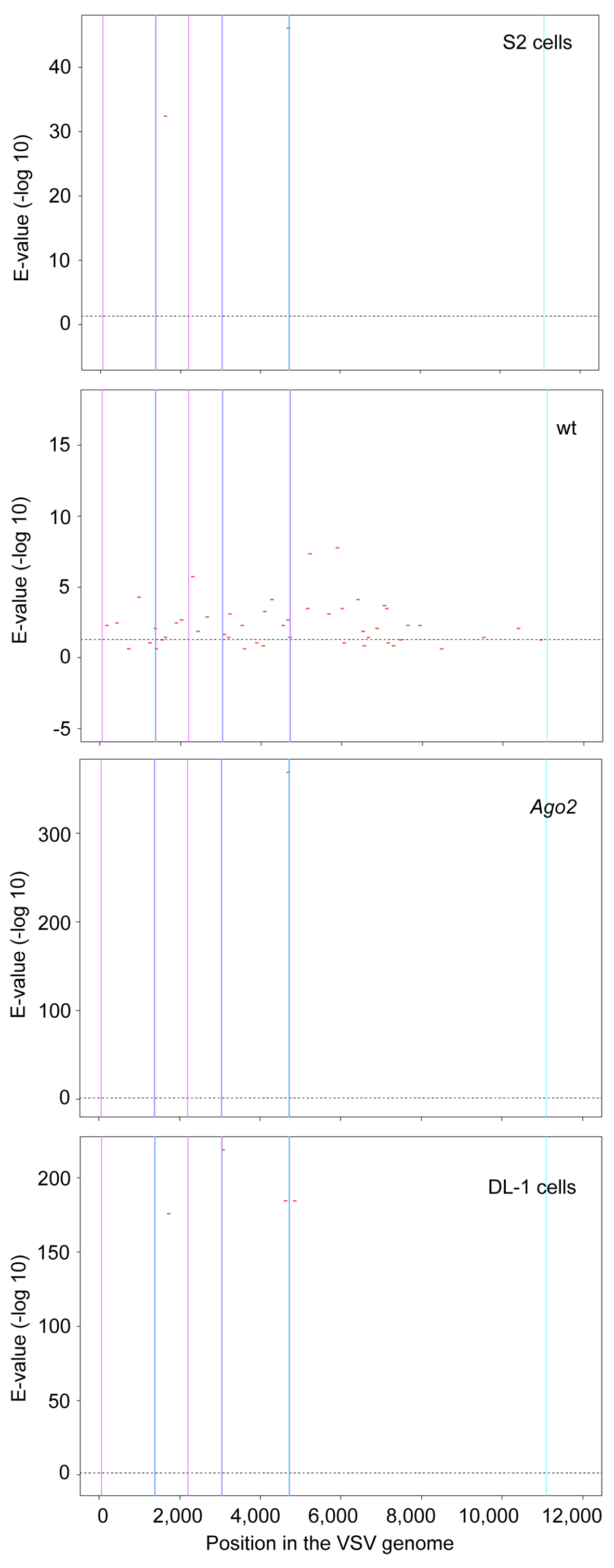

Supplement: Figure S5 — Analysis of gaps in vsiRNA coverage over the VSV genome as detected by independent sequencing experiments. Shown are the regions in the VSV genome in which no vsiRNAs were detected by high-throughput sequencing performed by Mueller et al [22] (S2 cells, wildtype (wt), and Ago2 mutants) and Sabin et al. [43] (DL-1 cells). These gaps in vsiRNA coverage are scaled to the genome. Vertical lines in each plot mark the gene promoters within the VSV genome. The probability that each gap did not occur by chance is shown as the inverse expected value (E-value) on a log10 scale. The horizontal line in each plot represents a significance cutoff of p = 0.05 that the gap occurred by chance. E-values above the line are even more significant. Note that the L gene promoter most consistently shows significant gaps in vsiRNA coverage. (TIF) [file ppat.1003579.s005.tif]

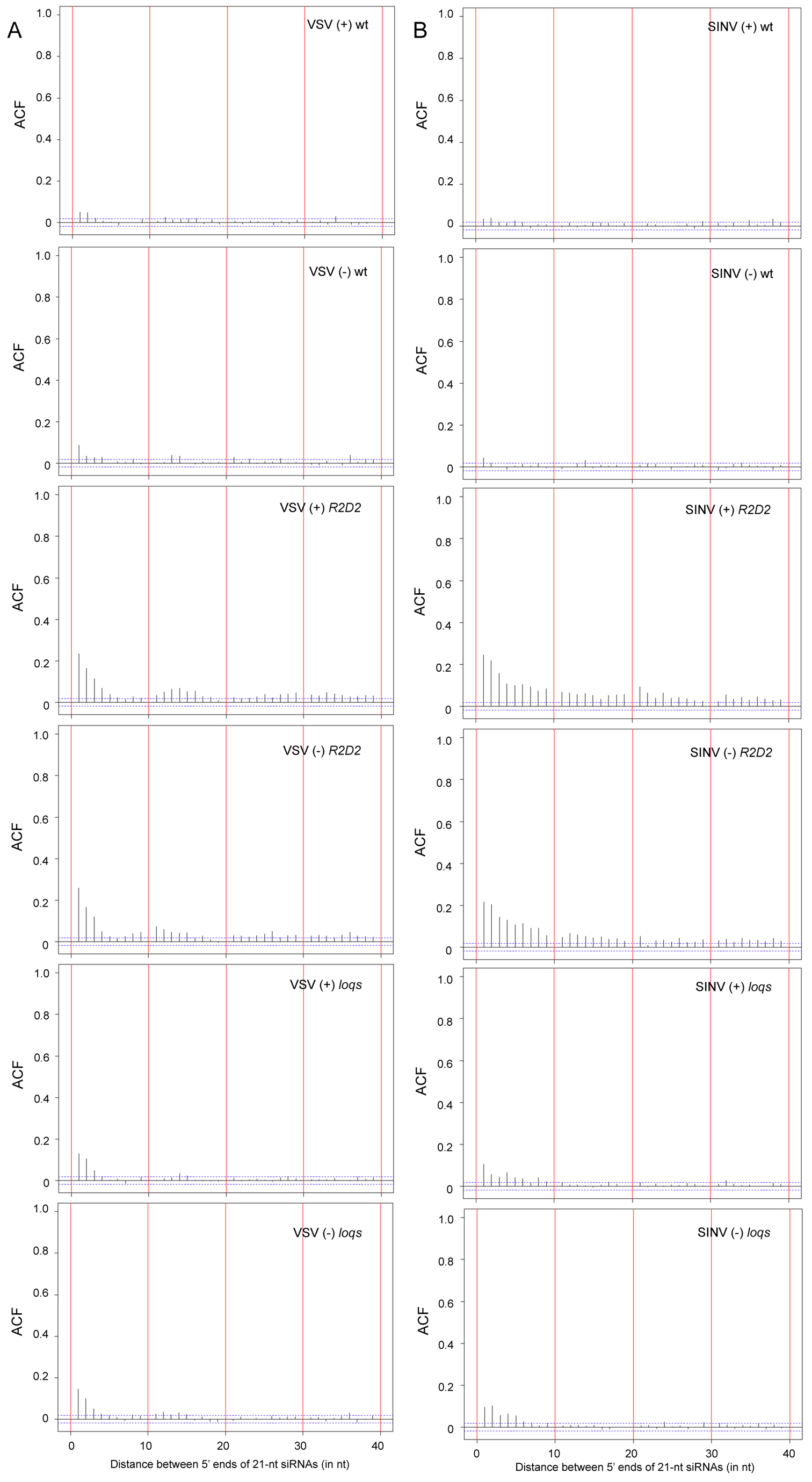

Supplement: Figure S6 — Phasing analysis of vsiRNAs derived from the positive and negative strands of SINV and VSV. Autocorrelation functions (ACF) of the distance in nucleotides between 5′ ends of vsiRNAs from VSV (A) and SINV (B) positive (+) and negative (−) strands, as indicated. The samples were generated from infected wildtype (wt), R2D2, and loqs mutants. ACF values above the dotted line are statistically significant (p<0.05). (TIF) [file ppat.1003579.s006.tif]

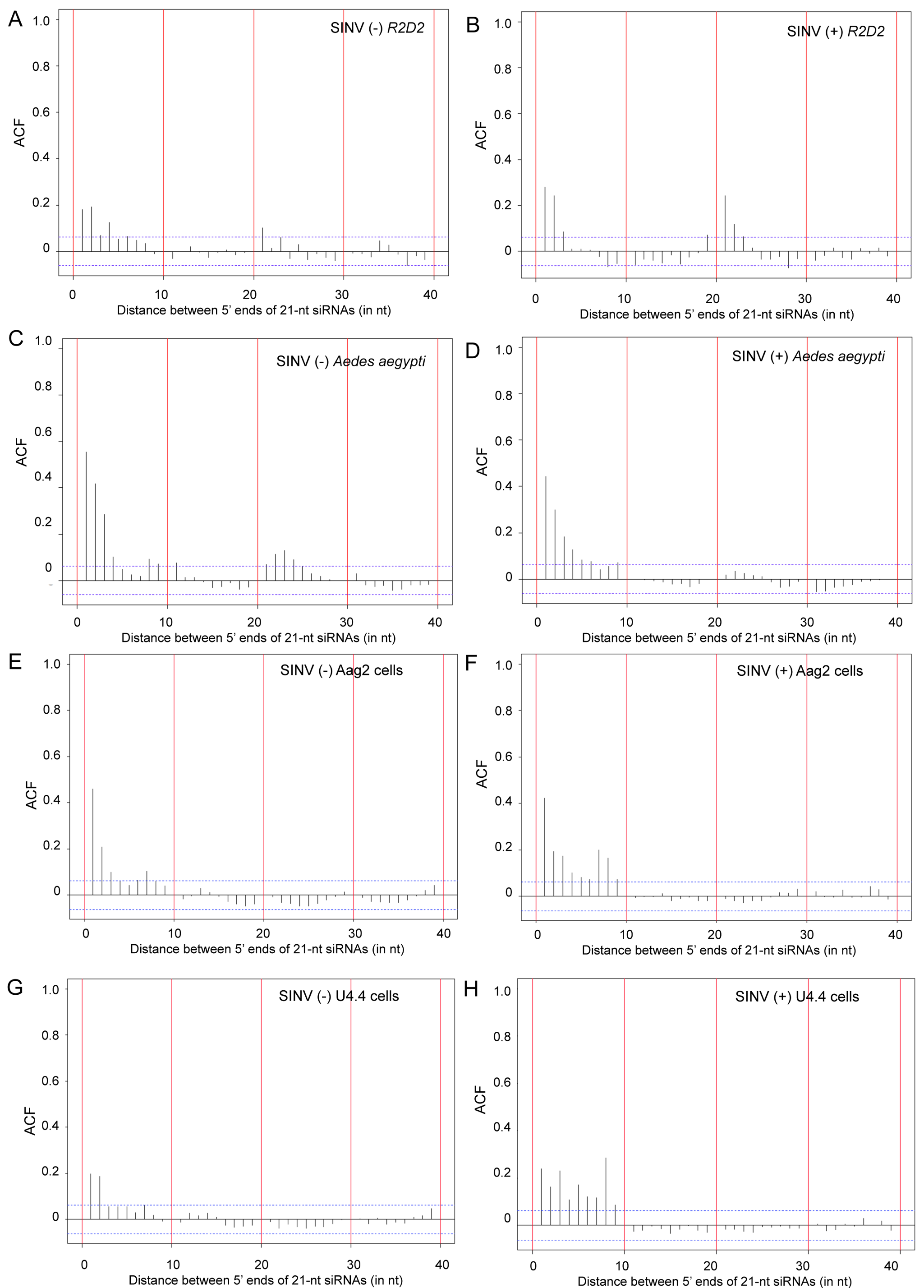

Supplement: Figure S7 — Phasing analysis of vsiRNAs derived from the SINV genome after infection of mosquitoes and Drosophila . Autocorrelation functions (ACF) of the distance in nucleotides between 5′ ends of vsiRNAs from SINV positive (+) and negative (−) strands. Shown are all vsiRNAs mapping to the 5′-most 1000 nts of the relevant strand. Samples were from our R2D2 mutant Drosophila (A,B), mosquitoes from Myles et al [40] (C,D), the cell line Aag2 (E,F), and cell line U4.4 (G,H) from Vodovar et al [48]. ACF values above the dotted line are statistically significant (p<0.05). (TIF) [file ppat.1003579.s007.tif]
