# Supplementary material for: Functional Specialization of the Small Interfering RNA Pathway in Response to Virus Infection
Source: PLoS Pathog. 2013 Aug 29;9(8):e1003579. doi: 10.1371/journal.ppat.1003579 (PMC3757037; doi:10.1371/journal.ppat.1003579)
Supplement: Table S1 — Statistical analysis of the differences in median survival with viral infection of different Drosophila mutants. (PDF) [file ppat.1003579.s008.pdf]

Table S1: Statistical analysis of the differences in median survival to viral infection in different *Drosophila* mutants.

Within groups		
**Genotype**	**Treatment comparison**	***p* value**
R2D2/+	Control vs Mock	0.9610
	Mock vs SINV	0.6707
	Mock vs VSV	0.2659
R2D2	Control vs Mock	0.0495
	Mock vs SINV	0.0112
	Mock vs VSV	0.0000
loqs/+	Control vs Mock	0.0703
	Mock vs SINV	0.2549
	Mock vs VSV	0.0269
loqs	Control vs Mock	0.0707
	Mock vs SINV	0.3409
	Mock vs VSV	0.4444
loqs-R2D2	Control vs Mock	0.6224
	Mock vs SINV	0.6230
	Mock vs VSV	0.0022
**Between groups**		
**Genotype comparison**	**Treatment**	***p* value**
R2D2/+ vs R2D2	control	0.2698
	Mock	0.0240
	SINV	0.0033
	VSV	0.0003
loqs/+ vs loqs	control	0.1711
	Mock	0.0216
	SINV	0.3845
	VSV	0.9142
R2D2 vs loqs-R2D2	control	0.3255
	Mock	0.1928
	SINV	0.1598
	VSV	0.0784
loqs vs loqs-R2D2	control	0.6062
	Mock	0.9064
	SINV	0.0069
	VSV	0.0073
