# Supplementary material for: Functional Specialization of the Small Interfering RNA Pathway in Response to Virus Infection
Source: PLoS Pathog. 2013 Aug 29;9(8):e1003579. doi: 10.1371/journal.ppat.1003579 (PMC3757037; doi:10.1371/journal.ppat.1003579)
Supplement: Table S2 — Summary of the raw data from the sequencing of the small RNA libraries described. (PDF) [file ppat.1003579.s009.pdf]

Table S2: Summary of the raw numbers from the analysis of the libraries described in this manuscript *

		Accession number / Genotype / Virus						
		wildtype VSV	*Dcr-2* VSV	*R2D2* VSV	*loqs* VSV	wildtype SINV	*R2D2* SINV	*loqs* SINV
Drosophila	**Transposons**	270,004	143,855	258,526	394,908	785,990	438,317	428,773
	**rRNA**	272,942	734,810	1,044,725	757,782	2,138,283	2,711,555	3,184,455
	**miRNA**	1,658,683	1,705,365	1,838,877	2,381,020	14,249,539	18,752,818	11,928,073
	**repeat**	3,215,314	2,596,031	4,013,364	5,452,095	11,696,828	7,127,218	8,768,479
	**mRNA**	259,933	78,834	150,161	215,182	895,951	656,939	387,897
	**No features**	598,262	346,507	499,296	813,823	2,302,877	2,029,849	1,617,725
	**Total mapped**	5,819,185	4,780,693	6,603,072	8,944,210	29,252,701	28,659,279	22,793,038
**Virus**	**VSV**	1,812	576	19,832	7,937	1	1	0
	**SINV**	0	0	1	5	3,463	20,143	5,612
	**Unmapped**	35,367,429	69,212,383	62,627,769	59,910,280	64,490,603	58,881,128	70,411,133
	**Total**	47,461,752	79,598,478	77,035,790	78,869,300	125,812,772	119,257,103	119,519,573

*Number of total reads in the library assigned per feature in the *Drosophila* genome or to VSV and SINV genomes. Reads >16 nt were considered.
