# Supplementary material for: Functional Specialization of the Small Interfering RNA Pathway in Response to Virus Infection
Source: PLoS Pathog. 2013 Aug 29;9(8):e1003579. doi: 10.1371/journal.ppat.1003579 (PMC3757037; doi:10.1371/journal.ppat.1003579)
Supplement: Table S3 — Percentage of reads in each feature class for the sequenced libraries. (PDF) [file ppat.1003579.s010.pdf]

Table S3: Percentage of reads in each feature class for the libraries described in this manuscript *

	wildtype	*Dcr-2*	*R2D2*	*loqs*	wildtype	*R2D2*	*loqs*
	**VSV**	**VSV**	**VSV**	**VSV**	**SINV**	**SINV**	**SINV**
**Transposon**	4.640	3.009	3.915	4.415	2.687	1.529	1.881
**rRNA**	4.690	15.370	15.822	8.472	7.310	9.461	13.971
**miRNA**	28.504	35.672	27.849	26.621	48.712	65.434	52.332
**repeats**	55.254	54.302	60.780	60.957	39.985	24.869	38.470
**mRNA**	4.467	1.649	2.274	2.406	3.063	2.292	1.702
**No features**	10.281	7.248	7.562	9.099	7.872	7.083	7.097
**VSV**	0.031	0.012	0.300	0.089	0.000	0.000	0.000
**SINV**	0.000	0.000	0.000	0.000	0.012	0.070	0.025

* Percentage of reads in each feature calculated relative to the total of mapped reads. Mapped reads >16 nt were considered. Unmapped reads are not included.
