# Supplementary material for: Functional Specialization of the Small Interfering RNA Pathway in Response to Virus Infection
Source: PLoS Pathog. 2013 Aug 29;9(8):e1003579. doi: 10.1371/journal.ppat.1003579 (PMC3757037; doi:10.1371/journal.ppat.1003579)
Supplement: Table S4 — Statistical analysis of C-enrichment and U-depletion at the first position of 21-nt vsiRNA sequence reads (PDF) [file ppat.1003579.s011.pdf]

Table S4: Statistical analysis of C-enrichment and U-depletion at the first position of 21-nt reads

	**C-enrichment at position 1 in the 5' end**					
**Virus**	VSV					
**Genotype**	wildtype		*R2D2*		*loqs*	
**Genome strand**	plus	minus	plus	minus	plus	minus
***p* value (vs genome composition)**	4.96E-08	4.50E-05	**0.374597356**	2.36E-04	1.50E-11	1.68E-14
***p* value (vs read nt composition)**	3.47E-06	0.015226039	**0.999138769**	**0.980383249**	0.003972282	0.003742173
**Virus**	SINV					
**Genotype**	wildtype		R2D2		loqs	
**Genome strand**	plus	minus	plus	minus	plus	minus
***p* value (vs genome composition)**	3.59E-05	1.79E-08	**0.241676722**	1.53E-10	0.000162761	0.00E+00
***p* value (vs read nt composition)**	0.002804033	5.78E-06	**0.955041929**	0.000356005	0.030168561	1.10E-11

	**U depletion at position 1 in the 5' end**					
**Virus**	VSV					
**Genotype**	wildtype		*R2D2*		*loqs*	
**Genome strand**	plus	minus	plus	minus	plus	minus
***p* value (vs genome composition)**	2.38E-10	3.49E-06	1.95E-18	4.21E-18	4.00E-13	4.32E-20
***p* value (vs read nt composition)**	3.12E-08	0.001503414	1.29E-11	2.45E-10	4.31E-13	6.18E-20
**Virus**	SINV					
**Genotype**	wildtype		R2D2		loqs	
**Genome strand**	plus	minus	plus	minus	plus	minus
***p* value (vs genome composition)**	0.049606029	0.038331565	**0.997226278**	**0.979822358**	**0.74128436**	0.034639679
***p* value (vs read nt composition)**	**0.167905594**	**0.053154857**	**0.999999992**	**0.999955092**	**0.824744834**	0.008304462

Values above 0.05 are highlighted in bold.
