# Supplementary material for: Functional Specialization of the Small Interfering RNA Pathway in Response to Virus Infection
Source: PLoS Pathog. 2013 Aug 29;9(8):e1003579. doi: 10.1371/journal.ppat.1003579 (PMC3757037; doi:10.1371/journal.ppat.1003579)
Supplement: Table S5 — Pairwise correlation of vsiRNA density along viral genomes (PDF) [file ppat.1003579.s012.pdf]

Table S5: Pairwise correlation of vsiRNA density along viral genomes

VSV antigenome			
	wildtype	*R2D2*	*loqs*
wildtype	1.0000000	0.3568898	0.3934236
R2D2	0.3568898	1.0000000	0.5839180
loqs	0.3934236	0.5839180	1.0000000
**VSV genome**			
	wildtype	*R2D2*	*loqs*
wildtype	1.0000000	0.4267428	0.4265496
R2D2	0.4267428	1.0000000	0.6530834
loqs	0.4265496	0.6530834	1.0000000
**SINV genome**			
	wildtype	*R2D2*	*loqs*
wildtype	1.0000000	0.3878873	0.4133285
R2D2	0.3878873	1.0000000	0.5683794
loqs	0.4133285	0.5683794	1.0000000
**SINV antigenome**			
	wildtype	*R2D2*	*loqs*
wildtype	1.0000000	0.3504123	0.3534574
R2D2	0.3504123	1.0000000	0.5289832
loqs	0.3534574	0.5289832	1.0000000
