# Supplementary material for: Functional Specialization of the Small Interfering RNA Pathway in Response to Virus Infection
Source: PLoS Pathog. 2013 Aug 29;9(8):e1003579. doi: 10.1371/journal.ppat.1003579 (PMC3757037; doi:10.1371/journal.ppat.1003579)
Supplement: Table S6 — Comparative analysis of the SOLiD and Illumina libraries from this work and other published studies. (PDF) [file ppat.1003579.s013.pdf]

Table S6: Comparative analysis of SOLiD and Illumina libraries:

Original work	Organism	Sample	Platform	Virus	number of reads			% mapped	21-nt reads
					Total	Post-filtering	Mapped against the virus		
Mueller et al	*Drosophila melanogaster*	S2 cells	illumina	VSV	5,347,026	5,116,387	46,538	0.909	37,472
		wildtype	illumina	VSV	6,597,381	6,294,884	6,707	0.106	4,897
		Ago2[414] mutants	illumina	VSV	7,159,317	6,895,521	598,799	8.684	440,689
Myles et al	*Aedes aegypti*	wildtype	illumina	SINV	4,376,164	4,034,966	581,211	14.40	486,730
This work	*Drosophila melanogaster*	wildtype	SOLiD	VSV	39,377,073	25,902,134	2,461	0.0095	1,891
		R2D2 mutants	SOLiD	VSV	70,200,097	36,787,341	41,137	0.1118	27,106
		wildtype	SOLiD	SINV	96,488,223	66,619,853	3,544	0.0053	2,577
		R2D2 mutants	SOLiD	SINV	90,177,938	62,472,641	21,377	0.0342	15,219
