# Supplementary material for: Functional Specialization of the Small Interfering RNA Pathway in Response to Virus Infection
Source: PLoS Pathog. 2013 Aug 29;9(8):e1003579. doi: 10.1371/journal.ppat.1003579 (PMC3757037; doi:10.1371/journal.ppat.1003579)
Supplement: Table S7 — Pairwise correlation of vsiRNA density along the VSV genome between the libraries in this work and Mueller et al. [22] . (PDF) [file ppat.1003579.s014.pdf]

Table S7: Pairwise correlation of vsiRNA density along the VSV genomes in wildtype flies between the libraries in our study and Mueller et al.

	**Correlation**
**VSV antigenome**	0.08945145
**VSV genome**	0.1340179
