# Supplementary material for: Functional Specialization of the Small Interfering RNA Pathway in Response to Virus Infection
Source: PLoS Pathog. 2013 Aug 29;9(8):e1003579. doi: 10.1371/journal.ppat.1003579 (PMC3757037; doi:10.1371/journal.ppat.1003579)
Supplement: Table S8 — Oligonucleotides used in experimental analysis. (PDF) [file ppat.1003579.s015.pdf]

Table S8: Oligonucleotides

			**Genomic location**	**sequence**
**Strand specific RT**	**SINV**	**+ genome RT**	SINV 6362R	gagcacattttgtagcgtgttc
		**- genome RT**	SINV 196L	cgcatctggccagtaaactaa
	**VSV**	**+ genome RT**	VSV 6521L	ttcggaccaattccagagat
		**- genome RT**	VSV 10865R	gcaatttgcatagccaatca
**qPCR**	**SINV**	**Left Primer**	SINV 3097 - 3114	cgttcagctgcaagacca
		**Right Primer**	SINV 3144 - 3162	taccggccgtggctagtat
		**Left Primer***	SINV 11039 - 11057	aggtcaatgccccgtacat
		**Right Primer***	SINV 11078 - 11100	catgtactgtcgactcttggaga
	**VSV**	**Left Primer**	VSV 8765 - 8787	cctttagaagggaattggaagaa
		**Right Primer**	VSV 8804 - 8824	tctgccgacttgataggattg

* Oligonucleotides utilized for the detection of SINV RNA after embryo injections
